# High‐Density Atomic Level Defect Engineering of 2D Fe‐Based Metal‐Organic Frameworks Boosts Oxygen and Hydrogen Evolution Reactions

**DOI:** 10.1002/advs.202405936

**Published:** 2024-10-30

**Authors:** Xin Zhao, Shixun Wang, Yanhui Cao, Yun Li, Arsenii S. Portniagin, Bing Tang, Qi Liu, Peter Kasák, Tianshuo Zhao, Xuerong Zheng, Yida Deng, Andrey L. Rogach

**Affiliations:** ^1^ Department of Materials Science and Engineering, and Center for Functional Photonics (CFP) City University of Hong Kong 83 Tat Chee Avenue, Kowloon Hong Kong SAR 999077 P. R. China; ^2^ School of Materials Science and Engineering Tianjin University Tianjin 300072 P. R. China; ^3^ Center for Advanced Materials Qatar University Doha 2713 Qatar; ^4^ The Department of Electrical and Electronic Engineering The University of Hong Kong Hong Kong SAR 999077 P. R. China; ^5^ School of Materials Science and Engineering Hainan University Haikou 570228 P. R. China

**Keywords:** atomic level defect, Fe‐MOF, hydrogen evolution, oxygen evolution, water splitting

## Abstract

Electrocatalysts based on metal‐organic frameworks (MOFs) attracted significant attention for water splitting, while the transition between MOFs and metal oxyhydroxide poses a great challenge in identifying authentic active sites and long‐term stability. Herein, we employ on‐purpose defect engineering to create high‐density atomic level defects on two‐dimensional Fe‐MOFs. The coordination number of Fe changes from 6 to 4.46, and over 28% of unsaturated Fe sites are formed in the optimized Fe‐MOF. In situ characterizations of the most optimized Fe‐MOF_0.3_ electrocatalyst during the oxygen evolution reaction (OER) process using Fourier transform infrared and Raman spectroscopy have revealed that some Fe unsaturated sites become oxidized with a concomitant dissociation of water molecules, causing generation of the crucial *OH intermediates and Fe oxyhydroxide. Moreover, the presence of Fe oxyhydroxide is compatible with the Volmer and Heyrovsky steps during the hydrogen evolution reaction (HER) process, which lower its energy barrier and accelerate the kinetics. As a result, the optimized Fe‐MOF electrodes delivered remarkable OER (259 mV at 10 mA cm^-2^) and HER (36 mV at 10 mA cm^-2^) performance. Our study offers comprehensive understanding of the effect of phase transformation on the electrocatalytic process of MOF‐based materials.

## Graphical abstract

Purposeful defect engineering is employed to create high‐density atomic‐level defects on two‐dimensional Fe‐MOFs via partial ligand replacement, resulting in over 28% unsaturated Fe sites. In situ characterization proves that some Fe unsaturated sites become oxidized, causing generation of the crucial *OH intermediates and Fe oxyhydroxide, and thereby boosting its performance in oxygen and hydrogen evolution reactions.

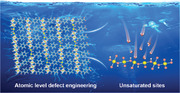

## 1 Introduction

Aiming to reduce CO_2_ emissions, hydrogen, especially green hydrogen, can play a tremendous role in replacing fossil fuels and ensuring a sustainable and low‐carbon energy economy.^[^ ^1^, ^2^ ^]^ Water electrolysis is a promising and environmentally friendly approach for generating high‐purity green hydrogen. At the present stage, water electrolysis is still impeded by high energy consumption due to the high overpotentials of oxygen and hydrogen evolution reactions (OER and HER, respectively). Thus, it is imperative to develop highly efficient and cost‐effective bifunctional electrocatalysts for both OER and HER to accelerate the kinetics of large‐scale hydrogen generation.

Metal‐organic frameworks (MOFs) have shown promising electrocatalytic activity toward OER and HER due to highly dispersed active sites, large surface area, high porosity, and component tunability.^[^ ^3^, ^4^, ^5^, ^6^, ^7^ ^]^ However, pristine MOFs often have low electroconductivity and a small exposure ratio of active sites and thus show non‐sufficient intrinsic electrocatalytic activity.^[^ ^3^, ^8^ ^]^ This is because the saturated coordination environment of active metal centers inhibits the efficient bonding of reactants, leading to a weak adsorption of the intermediates. Thus, low coordination numbers and unsaturated sites are crucial for MOFs‐based catalysts in order to tailor their electrocatalytic activity in OER and HER.^[^ ^9^, ^10^ ^]^ In recent years, atomic‐level catalysts with low coordination numbers and highly dispersed unsaturated sites have gained considerable attention in the field of electrochemical energy storage.^[^ ^11^, ^12^, ^13^, ^14^ ^]^ Several MOFs have been used as precursors for the production of atomic‐level catalysts. For instance, Müllen and co‐workers reported Co‐based catalysts derived from MOFs by high‐temperature pyrolysis, which exhibited a remarkable HER performance in an acidic solution, with an overpotential of 104 mV at 10 mA cm^−2^.^[^ ^15^ ^]^ However, MOFs‐based atomic‐level catalysts are inclined to decompose in acidic/alkaline electrolytes, while single metal atoms tend to aggregate to form metal nanoparticles during the catalytic reactions, which sacrifices the activity of the catalytic sites.^[^ ^16^ ^]^ A transition between MOFs and metal oxyhydroxide could occur during catalysis process, which would reconstruct the surface structure and introduce active sites. This dynamic change poses a great challenge in identifying authentic active sites and ensuring their long‐term stability. In addition, when fabricating membrane electrode assemblies, electrically insulating traditional binders such as Nafion and polyvinylidene fluoride can block active sites and impair the electrode conductivity.^[^ ^17^ ^]^ Therefore, it is crucial to further enhance the electroconductivity and intrinsic activity of MOF‐based electrocatalysts by breaking the saturated coordination environment and analyze the effect of the phase transformation process on water splitting.

To accelerate the kinetics of OER and HER, several strategies have been employed for MOFs to regulate the adsorption energy of the intermediates, such as doping heteroatoms,^[^ ^18^, ^19^ ^]^ as well as defect,^[^ ^20^ ^]^ interface,^[^ ^21^, ^22^ ^]^ and phase engineering.^[^ ^23^, ^24^ ^]^ Among them, defect engineering is an attractive approach to enhance the intrinsic catalytic activity while keeping the structural integrity of electrocatalysts.^[^ ^25^, ^26^, ^27^, ^28^, ^29^ ^]^ The presence of defects can induce electron delocalization, which would increase the electroconductivity and the charge density around the active sites.^[^ ^30^, ^31^ ^]^ Simultaneously, the coordination environment of the active sites can be modulated by the presence of defects to increase their exposure ratio and the active area, thus accelerating interactions between the reaction intermediates and catalytic sites.^[^ ^26^ ^]^ Different to the line, planar and volume defects, atomic defects would only distort the MOFs lattice on a short–range scale, thus resulting in a short‐range disorder while preserving the structural integrity and the overall stability of the catalyst.^[^ ^32^, ^33^ ^]^ But atomic defects are sensitive to the coordination environment, and it is a challenge to control and stabilize them.^[^ ^34^, ^35^ ^]^ It is crucial to suggest facile strategies to precisely control the type, density and distribution to achieve high‐density of atomic level defects. Overall, enabled by variable coordination structure of MOFs, defect engineering provides a feasible strategy to obtain electrocatalysts with both evenly distributed atomic vacancies (short‐range disorder) and structural integrity (long‐range order) at the same time.

Herein, we present a strategy to create high‐density atomic level defects on two‐dimensional (2D) Fe‐based MOFs.^[^ ^36^, ^37^ ^]^ Purposeful defect engineering in Fe‐MOFs was performed by partially substituting a multi‐coordinating bridging linker (1,4‐benzenedicarboxylic acid, BDC) with a non‐bridging ligand (benzoic acid, BA). The optimized Fe‐MOF electrocatalyst contained over 28% unsaturated Fe sites and possessed a short‐range disordered structure while preserving its structural integrity, as demonstrated by high‐angle annular dark‐field scanning transmission electron microscopy (HAADF‐STEM), X‐ray diffraction (XRD), Mössbauer spectra and transmission electron microscopy (TEM). The introduction of BA generated unsaturated sites, which regulated the electronic structure of Fe to a lower valence state and changed the coordination number of Fe atoms from 6 to 4.46, as proven by extended X‐ray absorption fine structure (EXAFS) measurements. Density functional theory (DFT) calculations showed that the presence of O vacancies optimized the adsorption of *O and H intermediates, thereby boosting the OER and HER performance of the optimized Fe‐MOF electrocatalyst, which showed OER and HER overpotentials of 259 and 36 mV at 10 mA cm^−2^ in 1.0 m KOH electrolyte, respectively. Moreover, in situ Fourier transform infrared (FTIR) and Raman spectroscopy studies performed on the most optimized Fe‐MOF_0.3_ electrocatalyst during the OER process have revealed H_2_O adsorption and crucial *OH intermediate production on the Fe unsaturated sites during OER process, which regulates the rate‐determining step of OER and thus boosts the OER activity and stability. Moreover, the conversion of Fe‐MOF_0.3_ to Fe oxyhydroxide regulated adsorption of H during the Volmer and Heyrovsky steps, which led to a significant improvement in the HER activity of Fe‐MOF_0.3_. The anion exchange membrane water electrolyzer based on the optimized Fe‐MOF electrocatalyst exhibited superior reversible durability under periodic potential (cycling between 1.6 to 1.9 V, 5 h per 0.1 V) for 125 h. Such high catalytic durability paves the way toward the realization of a hydrogen generation system that can be combined with renewable energy sources (e.g., wind, solar, tidal energy), which are random and volatile.

## 2 Results and Discussion

**Figure 1:**
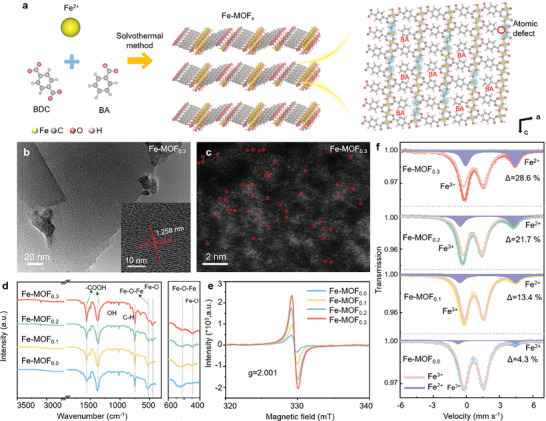
a) Schematic illustration of the fabrication of high‐density atomic defect Fe‐MOF_x_ electrocatalysts, utilizing combinations of BDC and BA linkers in different proportions, indicated by their ratio “x”. b) TEM image of a fragment of the Fe‐MOF_0.3_ catalyst; a characteristic lattice distance of Fe‐MOF_0.3_ equal to 1.258 nm is marked in the inset. c) HAADF‐STEM image of the surface of the Fe‐MOF_0.3_ catalyst; red circles indicate the locations of Fe atoms. d) FTIR spectra of the four studied Fe‐MOF_x_ catalysts; the panel on the right shows the magnified part of spectra in the range of 600–400 cm^−1^. e) EPR spectra and f) Mössbauer spectra of the four studied Fe‐MOF_x_ catalysts.

Crystalline Fe‐MOFs were synthesized through solvothermal reactions between Fe^2+^ ions and the multi‐coordinating bridging linker (BDC). Fe atoms are coordinated by six O atoms from BDC ligands and hydroxyls to form slightly distorted octahedral units, which extend to 2D layers by corner/edge sharing O. These 2D layers are separated by BDC ligands viewed from the *b* axis.^[^ ^38^, ^39^ ^]^ By using BA linkers to partially replace BDC in different proportions (see the Experimental Section of Supporting Information for details), BA ligands coordinate with the surface Fe atoms and generate tensile strain in the MOFs. This would also cause the formation of coordinatively unsaturated Fe sites on the exposed surfaces of the Fe‐MOFs, resulting in atomic‐level defects, as illustrated in **Figure** **1**a. In the forthcoming discussion, the obtained Fe‐MOFs will be denoted as Fe‐MOF_0.0_ (for those samples produced without any addition of BA) and Fe‐MOF_x_, where x represents the molar ratio of BA as compared to the total amount of ligands used (BDC and BA). Thus, four samples in total have been prepared with the molar ratio of BA changing from 0, 10, 20, to 30 (the most optimal one, as will be demonstrated below) mol%: Fe‐MOF_0.0_, Fe‐MOF_0.1_, Fe‐MOF_0.2_, and Fe‐MOF_0.3_, respectively. The morphology of these samples was studied by scanning electron microscopy (SEM), TEM, HADDF‐STEM, and XRD. As shown in Figure S1 (Supporting Information), 2D nanosheets self‐assembled to form three‐dimensional flower‐like structure in Fe‐MOF_0.0_, Fe‐MOF_0.1_, and Fe‐MOF_0.2_. With increasing the amount of BA as in Fe‐MOF_0.3_, the nanosheets became curlier and smaller, caused by increasing lattice strain. We notice that for the sample with the amount of BA further increased to 40%, a collapsed structure consisting of ill‐defined crystals was formed, as shown for Fe‐MOF_0.4_ in Figure S1e (Supporting Information), and thus, this amount of BA was not employed further. TEM images shown in Figure 1b (for Fe‐MOF_0.3_) and Figure S2 (Supporting Information) (for Fe‐MOF_0.0_, Fe‐MOF_0.1_, and Fe‐MOF_0.2_) further confirm the 2D nanosheet morphology of the four Fe‐MOFs. Due to the increasing degree of lattice distortion upon the increase of the BA amount, the characteristic lattice distance in Fe‐MOF_0.3_ (1.258 nm) estimated from TEM images was much larger than that of Fe‐MOF_0.0_ (1.198 nm), Fe‐MOF_0.1_ (1.206 nm) and Fe‐MOF_0.2_ (1.225 nm), which should indeed result in the anticipated (and desired) increased lattice strain in Fe‐MOF_0.3_. HAADF‐STEM measurements with sub‐angstrom resolution were employed to identify the locations of Fe atoms on 2D Fe‐MOFs nanosheets. As shown in Figure S3 (Supporting Information), after introducing the BA linker, bright white spots identifying positions of single Fe atoms (additionally marked with red circles) appeared in Fe‐MOF_0.1_ and Fe‐MOF_0.2_ due to the unsaturated Fe sites exposed at the surface of MOFs. Yet those spots were absent in the Fe‐MOF_0.0_ sample. As expected, the Fe‐MOF_0.3_ sample obtained with the largest amount of BA possessed the highest density of uniformly dispersed Fe single atoms (Figure 1c). Elemental mappings performed by the energy‐dispersive X‐ray (EDX) spectroscopy showed even distributions of Fe, C, and O atoms in all four composites (Figure S4, Supporting Information). The Fe contents are measured by inductively coupled plasma optical emission spectrometry (ICP‐OES) as 9.82, 10.16, 11.34, and 12.28 wt.% for Fe‐MOF_0.0_, Fe‐MOF_0.1_, Fe‐MOF_0.2_, and Fe‐MOF_0.3_, respectively.

The introduction of lattice strain in the BA‐derived Fe‐MOF_x_ has been further confirmed by the XRD patterns, as shown in Figure S5 (Supporting Information). All the characteristic diffraction peaks measured on the four studied Fe‐MOF_x_ catalysts align with the theoretical XRD simulated from the reference Fe‐MOF_0.0_ (CCDC‐1533883) shown as the line pattern at the bottom. The diffraction peak at 9.34° in Fe‐MOF_0.3,_ which belongs to the (200) plane, shifted downwards to 9.29°, 9.14°, and 8.91° for Fe‐MOF_0.1_, Fe‐MOF_0.2_, and Fe‐MOF_0.3_, respectively. From Bragg's equation, the interlayer spacing of the (200) plane is 9.46, 9.51, 9.66, and 9.91 Å, in Fe‐MOF_0.0_, Fe‐MOF_0.1_, Fe‐MOF_0.2_, and Fe‐MOF_0.3_, respectively, corresponding to the lattice expansion ratios of 0.5%, 2.0%, and 4.5% in the last three samples. XRD pattern confirmed that the long‐range order in the Fe‐MOF_0.3_ sample was maintained, even so, 30% BA has been added. Overall, more vacancies and larger lattice strains were created upon the introduction of BA into Fe‐MOFs, resulting in the high density of unsaturated Fe atoms (Figure 1c). N_2_ sorption isotherms (Figure S6, Supporting Information) were measured to determine the porosity of the four studied Fe‐MOF_x_ catalysts. The Brunauer–Emmett–Teller (BET) surface areas are calculated to be 812.5, 766.8, 749.6, and 634.8 m^2^ g^−1^ for Fe‐MOF_0.3_, Fe‐MOF_0.2_, Fe‐MOF_0.1_, and Fe‐MOF_0.0_, respectively, revealing partial substitution of the ligands increase the intrinsic porosities. The pore size distribution of Fe‐MOF_0.3_ was calculated using the Barrett–Joyner–Halenda desorption model, which shows peak maxima at 2.1 nm. Another small peak appeared at 3.93 nm which can be related to the defects around the unsaturated Fe atoms.

FTIR spectra of the four catalysts are shown in Figure 1d. The absorption peaks located at 1570 and 1380 cm^−1^ can be attributed to the asymmetric and symmetric vibrations of carboxyl groups (─COOH) of the BA and BDC, respectively.^[^ ^40^ ^]^ For the sake of comparison, the intensity ratio of absorption peaks at 1570 and 1380 cm^−1^ is plotted in Figure S7 (Supporting Information). The ratio $I_{1570\;cm^{-1}}/I_{1380\;cm^{-1}}$ changed from 0.55 to 0.92 with the BA ratio increasing from 0 to 30%, indicating the rise of the asymmetric vibration of ‐COOH around the Fe atoms. This again confirmed that BA replaces BDC ligands to coordinate with Fe atoms, as illustrated in Figure 1a, resulting in increased lattice strain, which regulates the coordination environment of Fe atoms. Moreover, the intensity of the peak at 522 cm^−1^ (see Figure 1d) assigned to the Fe─O─Fe vibrations decreased upon the introduction of BA, meaning that the amount of such bonds was reduced.^[^ ^41^ ^]^ In addition, the bending vibration of the Fe─O bond appeared at 447 cm^−1^, proving that Fe─O─Fe bonds broke and changed to Fe─O. Thermogravimetric analysis data of the four Fe‐MOF_x_ samples are shown in Figure S8 (Supporting Information). The first step of weight loss is due to the evaporation of ethanol solution and the bounded dimethylformamide. The second weight loss over the temperature range of 380–500 °C can be ascribed to the decomposition of Fe‐MOF_x_ as a result of the burning of organic‐linker molecules in the framework.

To determine the type of vacancies in Fe‐MOF_x_ samples, electron paramagnetic resonance (EPR) measurements were carried out. In Figure 1e, a Lorentz peak was observed at *g*  =  2.001 for all four Fe‐MOF_x_ samples, which could be attributed to the free electrons trapped by O vacancies.^[^ ^42^ ^]^ Among the four samples studied, Fe‐MOF_0.3_ showed the strongest EPR signal, confirming the presence of abundant O vacancies_,_ which can regulate the electronic structure of Fe atoms. To determine the oxidation state and the coordination environment of Fe atoms, Mössbauer spectroscopy was carried out. The Mössbauer spectra of Fe‐MOF_x_ derived from the recoil‐free absorption of γ‐rays by ^57^Co(Pd) nuclei could be deconvoluted into two different doublets (Figure 1f) according to the isomer shift (IS) and quadrupole splitting (QS) values which were assigned to high‐spin Fe^3+^ and high‐spin Fe^2+^, as summarized in Table S1 (Supporting Information).^[^ ^43^, ^44^, ^45^ ^]^ With the BA ratio increasing from 0 to 30%, the Fe^2+^ content increased from 4.3% to 28.6%, suggesting that using BA to replace BDC indeed resulted in changes of the electronic structure of Fe. Thus, the number of unsaturated sites in Fe‐MOF_0.3_ constitutes 28.6%. A much larger IS value of Fe^2+^ in Fe‐MOF_0.3_ suggested a higher delocalization of unpaired electrons, resulting in a larger 3d‐electron density of high‐spin Fe^2+^ in Fe‐MOF_0.3_, which would influence the overlap between the 3d orbital of Fe and antibonding π^*^orbital of O during the OER process.^[^ ^44^ ^]^ On the basis of the QS variation upon the increasing amount of BA (Table S1, Supporting Information), one can make a conclusion that BA induced an irreversible lattice distortion of the MOF structure around Fe, leading to a short‐range disordered structure. X‐ray photoelectron spectroscopy (XPS) survey was carried out to determine the chemical states of the constituting elements in Fe‐MOF_x_ catalysts (Figure S9, Supporting Information). The binding energy of Fe 2p_3/2_ (711.06 eV) in Fe‐MOF_0.3_ shifted to a lower value comparing with that of Fe‐MOF_0.2_ (711.25 eV), Fe‐MOF_0.1_ (711.46 eV), and Fe‐MOF_0.0_ (711.65 eV), proving that Fe in Fe‐MOF_0.3_ is in a lower valence state, which can be ascribed to the compensation of the overall charge imbalance caused by the deficiency of coordinative covalent bonds.

**Figure 2:**
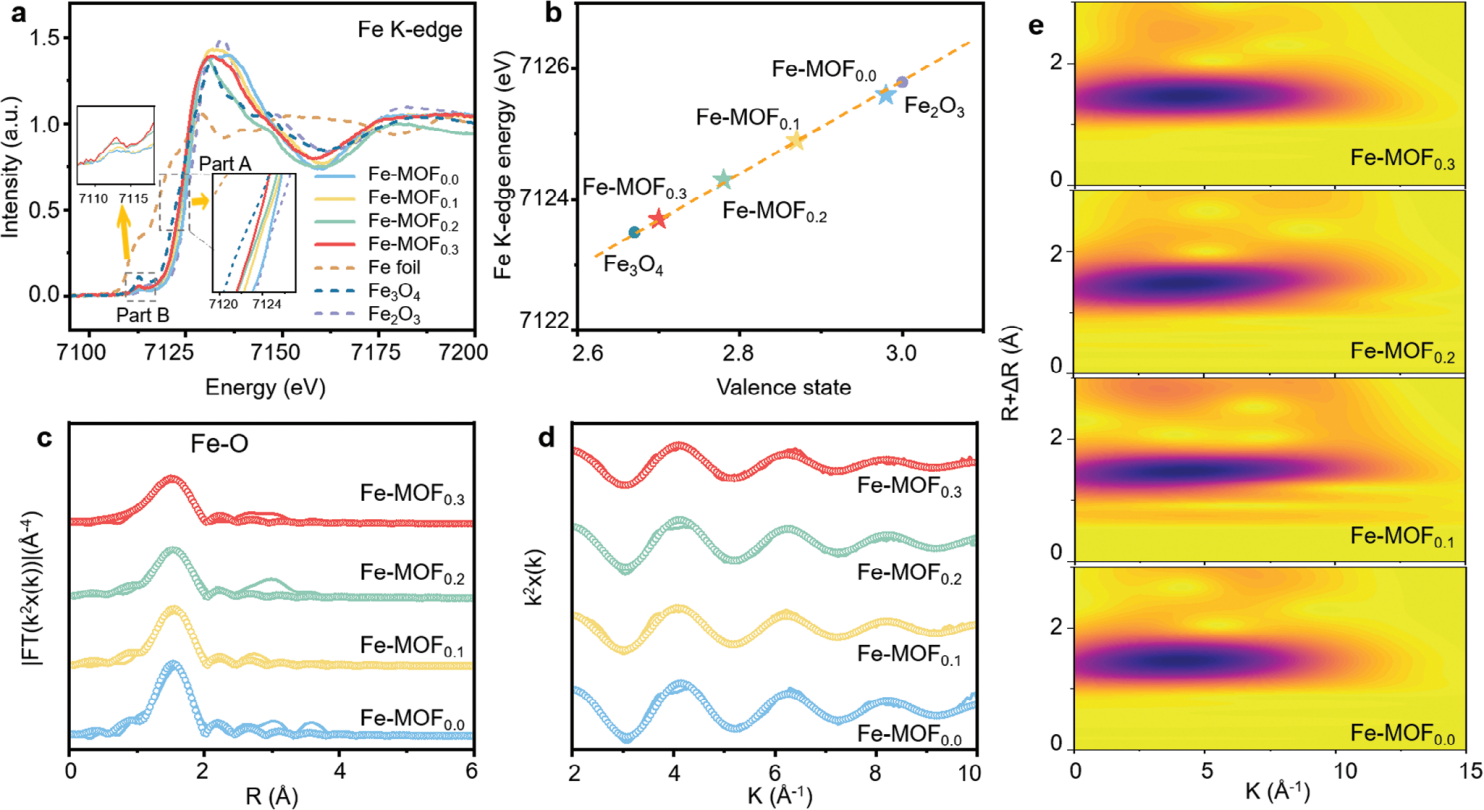
a) EXAFS spectra of Fe K‐edge for the four studied Fe‐MOF_x_ catalysts, with the reference spectra of Fe foil, Fe_3_O_4_, and Fe_2_O_3_ included for comparison. Two enlarged selected regions corresponding to Part A and Part B (see discussion in text) are shown in the insets. b) Valence states of Fe in the four studied catalysts, determined from the edge energy by averaging the linear rising edge of EXAFS spectra, as described in Experimental Section in Supporting Information. c) FT fitting curves and d) corresponding Re(*k* ^2^ *χ*(*k*)) oscillations of the four Fe‐MOF_x_ catalysts of the k^2^‐weighted Fe K‐edge EXAFS. e) MWT images of the Fe K‐edge EXAFS for the four Fe‐MOF_x_ catalysts.

Aiming to precisely determine the electronic valence state and the coordination environment of Fe atoms in the produced catalysts, we performed EXAFS measurements (**Figure** **2**a). Fe foil, Fe_2_O_3_, and Fe_3_O_4_ were used as Fe K‐edge references for calculating the valence states of Fe atoms in the four produced catalysts (Figure 2b, the detailed calculation method is presented in Experimental Section of Supporting Information). As shown in Figure 2a,b, the Fe K‐edge spectra of all four Fe‐MOF_x_ samples experienced a positive shift compared to Fe in Fe foil and Fe_3_O_4_ samples. From the magnitude of those shifts (Figure 2a Part A), we derived valence states of Fe atoms as equal to 2.70 in Fe‐MOF_0.3_, 2.78 in Fe‐MOF_0.2_, 2.87 in Fe‐MOF_0.1_, and 2.98 in Fe‐MOF_0.0_, respectively. These oxidation states of Fe are consistent with Mössbauer spectroscopy and XPS data presented in Figure 1f and Figure S9 (Supporting Information). The valence of Fe decreased when the amount of BA increased from 0 to 30%, suggesting that fewer electrons from Fe atoms transferred to surrounding O atoms. As shown in Part B of Figure 2a, higher intensity of the pre‐edge in Fe‐MOF_0.3_ proved that the structures of Fe‐MOF_x_ were distorted owing to the replacement of BDC by BA molecules, and most O vacancies were detected in Fe‐MOF_0.3_. To analyze the coordination structure of Fe atoms in Fe‐MOF_x_ catalysts, the least‐squares EXAFS fitting was performed, with related parameters shown in Figure 2c,d and Table S2 (Supporting Information). As compared with Fe─O and Fe‐Fe peaks in the reference samples (Figure S10, Supporting Information), the best‐fitting analysis in Fourier transform (FT) curves of k^2^‐weighted Fe K‐edge EXAFS (Figure 2c) showed one prominent peak corresponding to Fe─O (1.52 Å) in all the four catalysts, proving a similar coordination environment for Fe atoms. Notably, Fe‐MOF_0.3_ exhibited the least amplitude oscillations (Figure 2c), indicating that the average coordination number of Fe atoms decreased after introducing BA. The curve fitting revealed that the coordination number of the Fe─O bond in Fe‐MOF_0.3_ was 4.46, which is smaller than that in Fe‐MOF_0.2_ (4.88), Fe‐MOF_0.1_ (5.35), and Fe‐MOF_0.0_ (5.95), respectively. Such a smaller coordination number of Fe in Fe‐MOF_0.3_ should be related to the formation of unsaturated sites. To further substantiate this interpretation, we performed Morlet wavelet transform (MWT) analysis for four Fe‐MOF_x_ catalysts on the k^2^‐weighted EXAFS. MWT images of the Fe foil, Fe_3_O_4_, and Fe_2_O_3_ used as references are provided in Figure S11 (Supporting Information). As shown in Figure 2e, only one maximum was observed in the MWT images of Fe in all four Fe‐MOF_x_ catalysts, corresponding to the first coordination shell of O atoms. Overall, EXAFS data suggest that the introduction of BA generated O defects and Fe unsaturated sites in the Fe‐MOF_x_, which regulated the electronic structure and coordination numbers of Fe atoms in the studied catalysts.

**Figure 3:**
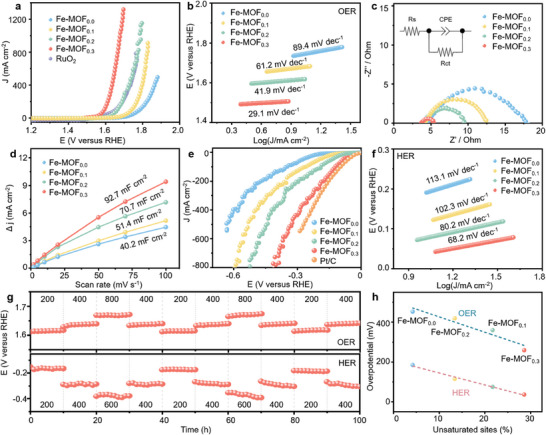
a) OER LSV curves of the four Fe‐MOF_x_ and RuO_2_ electrodes at 5 mV s^−1^. b) Tafel slopes of the four Fe‐MOF_x_ electrodes in OER. c) EIS Nyquist plots of the four Fe‐MOF_x_ electrodes, with the corresponding equivalent circuit shown in the inset. d) C_dl_ values of the four Fe‐MOF_x_ electrodes. e) HER LSV curves of the four Fe‐MOF_x_ and Pt/C electrodes at 5 mV s^−1^. f) Tafel slopes of the four Fe‐MOF_x_ electrodes in HER. g) OER chronopotentiometry response of Fe‐MOF_0.3_ electrodes at current densities of 200, 400, 800 mA cm^−2^, and HER chronopotentiometry response of Fe‐MOF_0.3_ electrodes at current densities of 200, 400, 600 mA cm^−2^. h) The relationship between the OER and HER overpotentials at a current density of 10 mA cm^−2^ and unsaturated site ratio of the four Fe‐MOF_x_ electrocatalysts obtained from Mössbauer spectra.

The electrocatalytic activities of the four studied Fe‐MOF_x_ catalysts in OER were evaluated using a standard three‐electrode device with N_2_ saturated 1.0 m KOH as electrolyte. Commercial RuO_2_ has been used as a reference electrode under identical conditions. As shown by the linear sweep voltammetry (LSV) curves presented in **Figure** **3**a and S12 (Supporting Information), the OER overpotential decreased significantly from 453 to 259 mV (10 mA cm^−2^) when the BA amount in catalysts increased from 0 to 30%; the latter value was superior to that of the commercial RuO_2_ (308 mV). For practical applications, the performance of catalysts under high current density is vitally important. As can be seen in Figure 3a, Fe‐MOF_0.3_ electrodes showed excellent electrocatalytic performance for OER with an overpotential of 470 mV even at a large current density of 1300 mA cm^−2^. The corresponding Tafel slopes (Figure 3b) also decreased from 89.4 to 29.1 mV dec^−1^ upon increasing the BA amount from 0 to 30%, demonstrating that the OER catalytic kinetics of Fe‐MOF_0.3_ electrodes were optimized through increasing the number of vacancies and lattice strain. Electrochemical impedance spectra (EIS) of the four Fe‐MOF_x_ electrodes were measured to evaluate their charge transfer resistance. The results shown in Figure 3c demonstrate that Fe‐MOF_0.3_ displayed the fastest charge transfer process among the four Fe‐MOF_x_ electrodes. The electrochemical active surface area (ECSA) of electrocatalysts is given by the double‐layer capacitance (*C*_dl_), which can be obtained from the CV curves at different sweep scan rates, provided in Figure S13 (Supporting Information) for all four studied electrodes. As shown in Figure 3d, Fe‐MOF_0.3_ had the highest *C*_dl_ (92.7 mF cm^−2^) among the four catalysts because more exposed active sites were utilized in the OER in this case. A chronopotentiometry test was applied to evaluate the operational stability; all four Fe‐MOF_x_ electrodes showed outstanding stability of the chronopotentiometry response with no evident decay for 150 h (Figure S14a, Supporting Information). Furthermore, the EPR curves (Figure S14b, Supporting Information) and ICP‐OES data (Table S3, Supporting Information) of the Fe‐MOF_0.3_ catalyst measured before and after its continuous operation 150 h also demonstrate the superior durability and excellent stability.

Moreover, the Fe‐MOF_0.3_ electrode also exhibited outstanding HER performance, as demonstrated in Figure 3e and Figure S15 (Supporting Information). It only required an overpotential of 36 mV to deliver a current density of 10 mA cm^−2^. At a high current density of 800 mA cm^−2^, Fe‐MOF_0.3_ showed an overpotential of 398 mV, outperforming that of commercial Pt/C and the other three Fe‐MOF_x_. Moreover, the Tafel slope (Figure 3f) of the Fe‐MOF_0.3_ (68.2 mV dec^−1^) was much smaller than that of Fe‐MOF_0.2_ (80.2 mV dec^−1^), Fe‐MOF_0.1_ (102.3 mV dec^−1^), and Fe‐MOF (113.1 mV dec^−1^), confirming its superior HER catalytic kinetics in the alkaline electrolyte. All four Fe‐MOF_x_ electrodes displayed long‐term stability over 150 h at 10 mA cm^−2^ (Figure S16, Supporting Information). The OER and HER catalytic stability of Fe‐MOF_0.3_ electrodes was also evaluated at different current densities, including 200, 400, and 800 mA cm^−2^ for OER and 200, 400, and 600 mA cm^−2^ for HER, respectively. As shown in Figure 3g, the Fe‐MOF_0.3_ electrode maintained the cycle stability under different current densities for 100 h, verifying its outstanding durability and excellent rate performance in catalyzing both OER and HER. This is an important advantage because it should be possible to combine such electrodes with unsteady‐current power supply systems such as wind, tidal, and solar to achieve green hydrogen production. As shown in Figure S17 (Supporting Information), the OER and HER performance of the Fe‐MOF_0.4_ electrode (with the content of BA equal to 40 mol%) were also evaluated. The overpotentials of the Fe‐MOF_0.4_ electrode in OER and HER were larger than Fe‐MOF_0.3_, because as mentioned above in relation to Figure S1e (Supporting Information), such higher content of BA destroyed the structural integrity of the Fe‐MOF, thus negatively influencing the mass and electron transfer.

The electrocatalytic performances of MOF‐based catalysts reported in recent literature are compared in Tables S4 and S5 (Supporting Information) for OER and HER, respectively. One can see that the Fe‐MOF_0.3_ catalyst developed here outperformed most of the reported catalysts. Figure 3h shows the relationship between the ratio of the unsaturated sites of Fe‐MOF_x_ catalysts (determined from their Mössbauer spectra provided in Figure 1f) and the overpotentials required to achieve a current density of 10 mA cm^−2^. Nearly linear correlation observed indicates the critical role of unsaturated sites in enhancing the intrinsic activity of the electrocatalyst. Overall, the Fe‐MOF_0.3_ catalyst with the highest amount of atomic‐level defects/exposure sites and efficient active area exhibited the best intrinsic catalytic activity in OER and HER.

**Figure 4:**
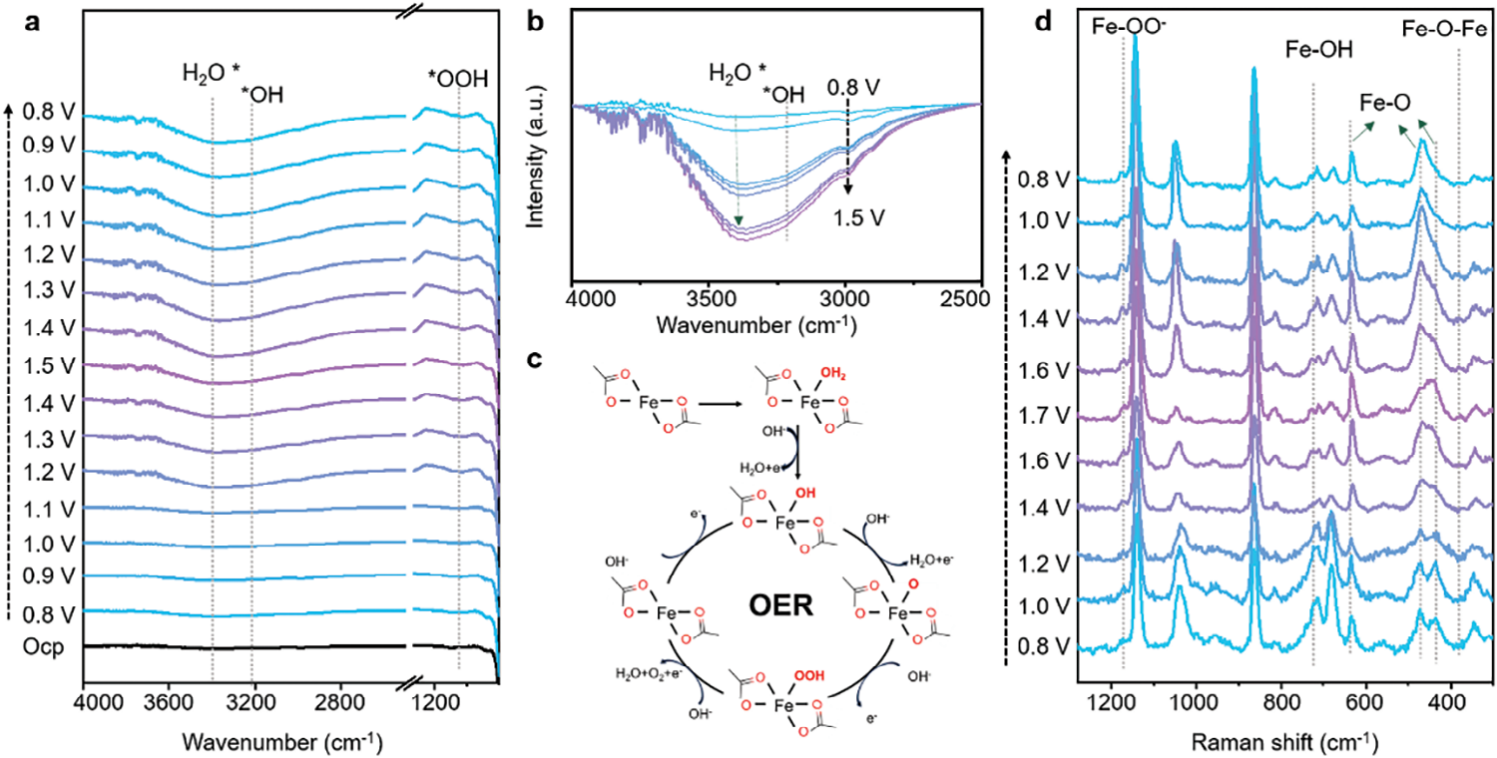
a) In situ FTIR spectra recorded under various potentials (vs RHE) which were performed on the Fe‐MOF_0.3_ electrocatalyst during the OER process. b) A detailed comparison of selected FTIR spectra recorded under potentials changing from 0.8 to 1.5 V. c) Proposed electron transfer OER pathway, and the corresponding pre‐OER Fe redox step for Fe‐MOF_0.3_. d) In situ Raman spectra recorded in the range of 1300 – 300 cm^−1^ under various potentials (vs RHE) for Fe‐MOF_0.3_ electrocatalyst during the OER process. All spectra were recorded during steady‐state conditions; the laser excitation wavelength was 532 nm, and the solvent was 1.0 m KOH.

To understand the OER mechanism of Fe‐MOF_0.3_ electrocatalyst, we performed in situ FTIR and Raman spectroscopy measurements (at different applied potentials versus RHE) to detect the reaction intermediates. Photographs of the two testing systems are shown in Figures S18 and S19 (Supporting Information), respectively. On the in situ FTIR spectra presented in **Figure** **4**a, the band at 3400 cm^−1^ which can be assigned to adsorbed water molecules (H_2_O^*^) shifted to 3209 cm^−1^ when the potential (vs RHE) varied from 1.0 to 1.5 V.^[^ ^46^ ^]^ In the detailed FTIR spectra presented in Figure 4b, the intensity of the vibration of H_2_O^*^ showed a positive correlation with applied potential. Similarly, the band at 3209 cm^−1^ assigned to OH* became more obvious after 1.0 V (Figure 4b), indicating that generated OH* comes from water dissociation as illustrated in Figure 4c.^[^ ^47^ ^]^ The large amount of *OH on Fe‐MOF_0.3_ is inclined to deprotonate to form OH^−^ (a hard Lewis base) as the bias voltage increases, with the local alkaline reaction environment formed facilitating the kinetic of both OER and HER.^[^ ^46^ ^]^ At the same time, the FTIR peak at 1092 cm^−1^ corresponding to OOH^−^ (Figure 4a) indicates the presence of Fe oxyhydroxide formed during OER process.^[^ ^48^ ^]^ In situ Raman spectra presented in Figure 4d point out the surface reconstruction of the Fe‐MOF_0.3_ electrocatalyst during OER process. Raman bands at 437, 469, and 634 cm*^−^* ^1^ can be assigned to the Fe─O bond with symmetric vibration (F_2g_), asymmetric bending (T_2g_) and symmetric stretching (A_1g_), respectively.[^49^, ^50^] Their Raman intensities are sensitive to structural disorders, such as defects and coordination environment.^[^ ^51^ ^]^ The intensity of the Fe‐O bond with F_2g_ mode decreases as the bias voltage increases, suggesting transformation of a part of Fe atoms.^[^ ^52^ ^]^ The intensities of the above‐mentioned Raman bands related to Fe‐O bond are summarized in Table S6 (Supporting Information). The relative intensity ratio of $I_{437\;cm^{-1}}/\left(I_{437\;cm^{-1}+}I_{469\;cm^{-1}+}I_{634\;cm^{-1}}\right)$ at the open circuit potential (Ocp) is 29.1%. This value is very similar to the proportion of Fe unsaturated sites previously determined by Mössbauer spectroscopy (28.6%), suggesting that the unsaturated Fe sites belong to F_2g_ vibration mode. Raman bands at 385, 728, and 1179 cm^−1^ can be ascribed to the Fe─O─Fe symmetric stretching, Fe─OH asymmetric stretching, and O─O top‐mode stretching vibrations of Fe oxyhydroxide, respectively, confirming the existence of the Fe oxyhydroxide phase.^[^ ^49^, ^53^, ^54^ ^]^ Hence, Fe atoms which were initially part of the Fe─O bond with the characteristic F_2g_ vibration mode transform into Fe oxyhydroxide, while the rest of the Fe atoms remain in the Fe‐MOFs structure, which prevents the structural collapse and improves the stability of the electrocatalyst.^[^ ^49^ ^]^ Moreover, when the applied potential increased to 1.6 V, a crucial intermediate *OH appeared and the intensity of its Raman signal became stronger with a further increase of the potential, but rapidly disappeared at 1.2 V. The dynamically appearing *OH suggests that the adsorption of *OH on the Fe active sites is a dynamic process upon changing potential. Raman spectrum of pristine Fe‐MOF_0.3_ was compared with that of the electrocatalyst after cycling it for 6 h, as shown in Figure S20 (Supporting Information). No obvious change of the Raman bands belonging to carboxylate linkers in the range of 600 to 1700 cm^−1^ was detected, indicating they would not experience leakage from the MOF structure, which again confirms the superior stability of this electrocatalyst.^[^ ^55^ ^]^ Combining these observations with Mössbauer and XAFS data, we conclude that the unsaturated Fe sites with a low coordination number can pre‐adsorb more water molecules (see Figure 4a). When the potential was applied, some amount of Fe becomes oxidized with a concomitant dissociation of water molecules, which results in the generation of the crucial *OH intermediates and the metal Fe oxyhydroxide; this promotes the kinetic of OER process.

In situ Raman spectra of Fe‐MOF_0.3_ were acquired to achieve understanding of the surface reconstruction during HER process. Figure S21a (Supporting Information) shows a series of Raman spectra for Fe‐MOF_0.3_ immersed in 1.0 m KOH at selected applied potentials versus RHE. The peak at 436 cm^−1^ assigned to F_2g_ vibrational modes of Fe─O bond decreased in intensity when the potential swept in the HER range of 0.2–−0.2 V. This confirmed that the Fe atoms forming the Fe─O bond with the characteristic F_2g_ vibration mode transformed into Fe oxyhydroxide. The reaction mechanism as shown in Figure S21b (Supporting Information) can be summarized as follows. Water molecules are adsorbed on the Fe site and dissociate to form the adsorbed H (H_ad_) intermediates on Fe sites (H_ad_–Fe) and Fe oxyhydroxide (Volmer step). Then, a similar water dissociation takes place on the H_ad_–Fe site, accompanied by H_2_ and Fe oxyhydroxide (Heyrovsky step). Fe oxyhydroxide helps to split the HO─H bond during the Volmer and Heyrovsky steps, thus regulating the adsorption energy of H_ad_ of the HER process in alkaline solution. Thus, transformation of Fe‐MOF_0.3_ in the HER process results in a higher charge transfer rate for this catalyst, consequently further reducing the reaction barrier and optimizing its catalytic activity.

**Figure 5:**
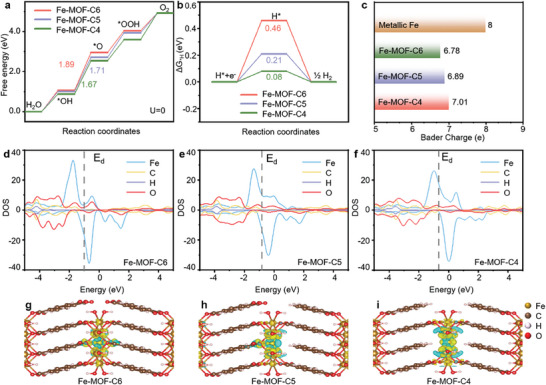
a) Calculated free energy diagrams (at an equilibrium potential of 0 V) showing overall OER pathways of the model Fe‐MOF‐Cx electrocatalysts. b) Calculated Gibbs free energies of hydrogen adsorbed on the model Fe‐MOF‐Cx electrocatalysts. c) Calculated Bader charges of metallic Fe, and of Fe single atoms on Fe‐MOF‐C_X_ catalysts. Calculated PDOS of the Fe *d*‐band, C *p*‐band, H *s*‐band, and O *p*‐band for d) Fe‐MOF‐C6, e) Fe‐MOF‐C5, and f) Fe‐MOF‐C4; the d‐band center is marked by dashed lines for each case. The charge density difference and the calculated Bader charges of active sites for g) Fe‐MOF‐C6, h) Fe‐MOF‐C5, and i) Fe‐MOF‐C4. Yellow and cyan colors indicate charge accumulation and depletion, respectively.

To unveil the catalytic enhancement mechanism of OER and HER occurring on Fe‐MOF_x_, we performed DFT calculations of adsorption energies and electronic structures of related model structures. For DFT calculations, Fe‐MOF_0.0_ structure was constructed by octahedrally coordinated divalent Fe and BDC. In the original 2D MOF (Fe‐MOF_0.0_), the coordination number of Fe is equal to six, and thus, we denote this structure as Fe‐MOF‐C6 (Figure S22, Supporting Information). By partially replacing BDC with an increasing amount of BA, the resulting two unsaturated Fe‐MOF_x_ models were named Fe‐MOF‐C5 and Fe‐MOF‐C4 (Figure S22, Supporting Information). As the next step, models of Fe‐MOF‐C6, Fe‐MOF‐C5, and Fe‐MOF‐C4 with adsorbed oxygen‐containing intermediates *O, *OH, and *OOH were established, as illustrated in Figure S23 (Supporting Information). In **Figure** **5**a, we provided Gibbs free energy diagrams of the three model Fe‐MOFs for OER, with Fe being the active site. The rate‐determining step (RDS) for the three model Fe‐MOFs is the transfer from *OH to *O. The calculated Gibbs free energy (ΔG) value of the RDS for Fe‐MOF‐C4 (1.67 eV) was much smaller than that of Fe‐MOF‐C5 (1.70 eV) and Fe‐MOF‐C6 (1.89 eV). When the coordination number changed from 6 to 4, the adsorption strength of *O decreased, thus promoting occurrence of OER. Gibbs free energies of the hydrogen adsorption (ΔG_H*_) for three model Fe‐MOFs were also calculated and are shown in Figure 5b. Fe‐MOF‐C4 exhibited lower value of ΔG_H*_ (0.08 eV) as compared to Fe‐MOF‐C5 (0.21 eV) and Fe‐MOF‐C6 (0.46 eV), suggesting the faster formation and release of the molecular hydrogen in this case. To understand the electron transfer process around Fe atoms coordinated with O, we further calculated Bader charges of the three model Fe‐MOFs and metallic Fe for comparison (Figure 5c). Bader charge decreased from 8 in the metallic Fe to 7.01 in Fe‐MOF‐C4, indicating that one electron was transferred to the surrounding O. This was less than in the case of Fe‐MOF‐C6 (1.22) and Fe‐MOF‐C5 (1.11), which means that Fe atoms in Fe‐MOF‐C4 are in a lower valence state as compared to Fe‐MOF‐C5 and Fe‐MOF‐C6.

Projected density of states (PDOS) was calculated to probe the electronic interaction between the adsorbate and three model Fe‐MOFs. PDOS of Fe 3d orbitals and that of the coordinated O 2p orbitals have a large overlap at the energy range of −5 to 5 eV, as shown in Figure 5d–f, suggesting that Fe and O in all three model Fe‐MOFs experienced strong hybridization. The antibonding states located above the Fermi level can describe the adsorbate‐metal interaction because the electron filling the antibonding state mainly contributes to the bond strength between the adsorbate valence states and the transition‐metal d states.^[^ ^56^ ^]^ Fe‐MOF‐C4 had its *d*‐band center at −0.652 eV as indicated by the grey dash line in Figure 5f, which was closer to the Fermi level as compared to Fe‐MOF‐C5 (−0.821 eV) and Fe‐MOF‐C6 (−0.997 eV). Thus, the antibonding energy states of MOF‐C4 shifted toward higher energy positions, and the interaction between the adsorbate and the active site was strengthened. Combined with ΔG value of the RDS, the upshift of the d‐band center of Fe‐MOF‐C4 can facilitate the adsorption energy of the H and *O for HER and OER, respectively.^[^ ^57^, ^58^, ^59^, ^60^, ^61^ ^]^ In addition, the total DOS of Fe‐MOF‐C4 around the Fermi energy level was larger than that of Fe‐MOF‐C5 and Fe‐MOF‐C6, which means that Fe‐MOF‐C4 should possess higher electroconductivity.^[^ ^62^ ^]^ The yellow and cyan areas in Figure 5g–i indicate the electron charge accumulation and depletion, respectively. The electronic charge distribution in Fe‐MOF‐C6 was symmetric, and the charges were accumulated around the O atoms owing to their high electronegativity (3.44) on the Pauling scale. The central Fe atom in Fe‐MOF‐C5 showed greater charge loss, especially at the end facing the BA linker. More electrons were accumulated at the Fe site in Fe‐MOF‐C4 as compared to Fe‐MOF‐C5 and Fe‐MOF‐C6, because of the less coordinated O atoms around the Fe site. Therefore, the electron‐sufficient atomic Fe site in Fe‐MOF‐C4 would facilitate the adsorption of intermediates and lower the energy barrier of RDS.

**Figure 6:**
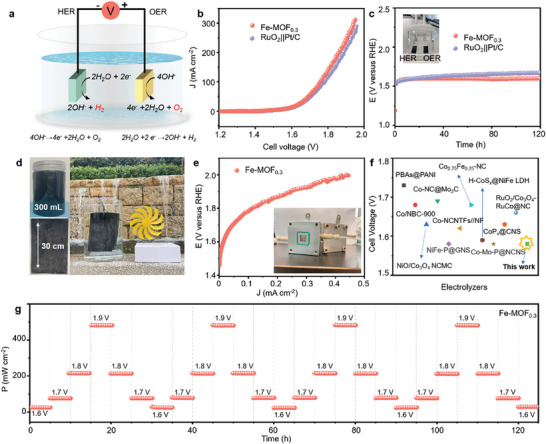
a) Illustration of the overall water splitting device. b) LSV responses of the water electrolyzers constructed from Fe‐MOF_0.3_ (+,‐) and RuO_2_(+)||Pt/C(‐) in 1.0 m KOH at a scan rate of 5 mV s^−1^. c) Potentiometry stability of the Fe‐MOF_0.3_ (+,‐) and RuO_2_(+)||Pt/C(‐) electrodes for a long‐term operation at an initial current density of 10 mA cm^−2^ in 1.0 m KOH; inset is a photograph of a two‐electrode electrolyzer device. d) Photographs of 300 mL slurry of Fe‐MOF_0.3_ (top picture), dry‐painted Fe‐MOF_0.3_ electrode on a Ni foil (bottom picture), and two‐electrode electrolyzer supplied by a small wind turbine. e) Polarization curves of the AEMWE based on Fe‐MOF_0.3_ (+,‐), with an inset showing the photograph of this device. f) Cell voltage comparison between the Fe‐MOF_0.3_ (+,‐) electrodes and previously reported MOFs‐based electrolyzers at a current density of 10 mA cm^−2^. g) Power‐time curve of the AEMWE based on Fe‐MOF_0.3_ (+,‐) periodically operated at 1.6, 1.7, 1.8, and 1.9 V swapped every 5 h.

Inspired by the outstanding OER and HER activity of the most optimized Fe‐MOF_0.3_ catalyst, the overall water splitting electrolyzer has been assembled while using it as both the anode and cathode electrode, to demonstrate the applicability of Fe‐MOF_0.3_ in practical water splitting systems (**Figure** **6**a). The potential polarization curves of Fe‐MOF_0.3_ and Pt/C||RuO_2_ water electrolyzer in 1.0 m KOH are shown in Figure 6b and Figure S24 (Supporting Information). Remarkably, the overall water‐splitting performance of Fe‐MOF_0.3_ (1.58 V) was superior to the Pt/C||RuO_2_ (1.61 V) at 10 mA cm^−2^. In addition, the excellent stability of Fe‐MOF_0.3_ measured at 10 mA cm^−2^ for 120 h is presented in Figure 6c, and is much better than that of Pt/C||RuO_2_. Vigorous gas emission could be observed from both electrodes on photographs given as insets in Figure 6c, when OER and HER occurred. Moreover, Fe‐MOF_0.3_ exhibited higher electrolysis energy efficiency of ≈93.6% at 10 mA cm^−2^ than that of the Pt/C||RuO_2_ electrocatalysts (91.9%) (the detailed calculation method is presented in Experimental in Supporting Information).

To put forward the practical large‐scale application of the Fe‐MOF_x_ catalyst, we made 300 mL slurry from Fe‐MOF_0.3_ and deposited it onto the Ni foil by spray painting to fabricate large‐scale electrodes, as shown by photographs in Figure 6d. These electrodes were applied in a two‐electrode electrolysis device powered by a small wind turbine (Figure 6d) to realize overall water splitting. We also fabricated a membrane electrode assembly using Fe‐MOF_0.3_ as both cathode and anode, and assembled an anion exchange membrane water electrolyzer (AEMWE) prototype, illustrated by the photograph shown in the inset of Figure 6e. Rather impressively, this AEMWE based on Fe‐MOF_0.3_ achieved industrial current densities of 400 mA cm^−2^ at a low cell voltage of 1.97 V. We also observed that the water‐splitting performance of Fe‐MOF_0.3_ based electrolyzer was better than recently reported MOF‐based catalysts, as summarized in Figure 6f and Table S7 (Supporting Information). The cyclic stability of the AEMWE has been evaluated by applying the potential periodically (cycling between 1.6 V to 1.9 V, 5 h per 0.1 V), thus achieving four cycles for 125 h in total, as shown in Figure 6g. The highest power reached 480 mW cm^−2^ at 1.9 V, suggesting that Fe‐MOF_0.3_ electrodes were robust under continuous operating conditions. Thus, it is indeed promising to combine them with renewable energy sources (e.g., wind, solar, hydro energy), which are random, intermittent, and volatile, to implement overall water splitting.

## 3 Conclusion

In summary, we have successfully realized a high‐density atomic‐level defect engineering on 2D Fe‐MOFs via partial ligand replacement. The defect engineering was favorable for manipulating the distribution density of the Fe atoms and redistributing the charge density. The introduction of 30 mol% of BA ligands generated over 28% unsaturated Fe sites, which regulated the electronic structure and coordination number of Fe atoms (changing from 6 to 4.46) and thus offered optimal electrocatalytic activity of the produced Fe‐MOF_0.3_ catalyst for HER and OER. As a result, Fe‐MOF_0.3_ electrodes delivered remarkable OER (259 mV at 10 mA cm^−2^) and HER (36 mV at 10 mA cm^−2^) performance. In situ FTIR and Raman measurements performed on the most optimized Fe‐MOF_0.3_ electrocatalyst during the OER process indicated that pre‐adsorption of water on the unsaturated Fe sites generated a crucial *OH intermediate, thus greatly accelerating the reaction kinetics. In situ Raman measurements performed during the HER process indicate that Fe oxyhydroxide can help to split the HO‐H bond during the Volmer and Heyrovsky steps, and thus regulate the adsorption energy of H_ad_ in the HER process in alkaline solutions. DFT calculations revealed that the better performance of Fe‐MOF_0.3_ electrodes can be attributed to the larger 3d‐electron density of Fe site, which facilitated the adsorption of *O and H and lowered the energy barrier of the RDS process. AEMWE based on Fe‐MOF_0.3_ electrodes displayed a high cyclic stability under continuous operating conditions for 125 h by applying the potential periodically (cycling between 1.6 to 1.9 V, 5 h per 0.1 V). Thus, it is promising to combine Fe‐MOF_0.3_ based electrolyzer with unsteady‐current power supply systems such as wind, tidal, or solar energy to achieve reliable green hydrogen production.

## Conflict of Interest

The authors declare no conflict of interest.

## Supporting information



Supporting Information

## Data Availability

The data that support the findings of this study are available in the supplementary material of this article.
