# Supplementary material for: High‐Density Atomic Level Defect Engineering of 2D Fe‐Based Metal‐Organic Frameworks Boosts Oxygen and Hydrogen Evolution Reactions
Source: Adv Sci (Weinh). 2024 Oct 30;11(47):2405936. doi: 10.1002/advs.202405936 (PMC11653667; doi:10.1002/advs.202405936)
Supplement: Supplementary file 1 — Supporting Information [file ADVS-11-2405936-s001.docx]

**Supporting Information**

**High-density atomic level defect engineering of two-dimensional Fe-based metal-organic frameworks boosts oxygen and hydrogen evolution reactions**

*Xin Zhao, Shixun Wang, Yanhui Cao, Yun Li, Arsenii S. Portniagin, Bing Tang, Qi Liu, Peter Kasák, Tianshuo Zhao, Xuerong Zheng,^*^* *Yida Deng, Andrey L. Rogach^*^*

X. Zhao, S. Wang, Y. Li, A. S. Portniagin, Dr. B. Tang, Q. Liu, Prof. A. L. Rogach

Department of Materials Science and Engineering, and Center for Functional Photonics (CFP), City University of Hong Kong, 83 Tat Chee Avenue, Kowloon, Hong Kong S.A.R. 999077, P. R. China

E-mail: andrey.rogach@cityu.edu.hk

Y. Cao

School of Materials Science and Engineering, Tianjin University, Tianjin, 300072, P. R. China

Prof. P. Kasák

Center for Advanced Materials, Qatar University, PO Box 2713, Doha, Qatar

Prof. T. Zhao

The Department of Electrical and Electronic Engineering, The University of Hong Kong, Hong Kong S.A.R. 999077, P. R. China

Prof. X. Zheng, Prof. Y. Deng

School of Materials Science and Engineering, Hainan University, Haikou, 570228, P. R. China

E-mail: xrzh@tju.edu.cn

**Experimental Section**

**Chemicals.** FeCl_2_·6H_2_O (99.99% AR grade), 1,4-benzenedicarboxylic acid (BDC), benzoic acid (BA), Nafion solution (5 wt%), KOH (99.98%), triethylamine (TEA) and *N*,*N*-dimethylformamide (DMF) were bought from Aladdin Reagent. All chemicals were used directly without further purification. Ultrapure water (18 M) used in the experiments was from a Millipore System (Millipore Q).

**Synthesis of Fe-MOF****_0.0_.** DMF (32 ml), ethanol (2 ml) and water (2 ml) were mixed in a 100 ml polytetrafluoroethylene (PE) tube, and 1 mmol BDC was dissolved in this mixture under ultrasonication. Subsequently, 1 mmol FeCl_2_·6H_2_O was dissolved, and 0.8 ml TEA was quickly injected. The mixture was stirred for 5 min to obtain a uniform colloidal suspension, and then continuously ultrasonicated for 8 h at 40 kHz under air-tight conditions. The product obtained was separated via centrifugation, washed with ethanol 3-5 times, and dried at room temperature.

**Synthesis of Fe-MOF_0.1_, Fe-MOF_0.2_, and Fe-MOF_0.3_.** The preparation process was the same as for Fe-MOF_0.0_ described above, except that 1 mmol BDC was replaced by {0.1, 0.2 or 0.3 mmol BA plus 0.9, 0.8 and 0.7 mmol BDC} for Fe-MOF_0.1_, Fe-MOF_0.2_, and Fe-MOF_0.3_ samples, respectively.

**Characterization.** SEM images were obtained on a FEI Quanta FEG 450 instrument. TEM, HRTEM, and HAADF-STEM images were collected on JEM-ARM200F. XRD patterns were measured on a D2 PHASER using Cu Kα radiation (λ = 0.154 nm). XPS spectra were recorded in an ultra-high vacuum using Al K_α_ X-ray source, and calibrated by C 1s binding energy (284.8 eV) on a VG ESCALAB 220i-XL instrument. FT-IR data were collected on a [PerkinElmer FTIR spectrometer](https://www.cityu.edu.hk/mse/studentlan/equip/B2640_FTIR.jpg). Raman spectra were measured on a [WITec alpha 300R Raman System](https://www.cityu.edu.hk/mse/studentlan/equip/R7167_Raman.jpg). Mössbauer spectra were measured on a proportional counter with Germany Wissel accelerator at room temperature. The radioactive source was ^57^Co(Pd), and the spectra were fitted by the least square method. XAS measurements for the Fe K-edge were conducted in transmission mode on the beamline 12-BM of the Advanced Photon Source at the Argonne National Laboratory. Fe K-edge EXAFS data were processed according to the standard procedures using the ATHENA module implemented in the IFEFFIT software package.^[1-2]^ After subtracting the post-edge background from the overall absorption and normalization with respect to the edge jump step, EXAFS spectra were obtained. To determine the valence states of Fe in the catalysts, a nearly linear portion of the rising edge was selected for integration, and integrated average intensity was defined as the K-edge. Similar method was used to determine the K-edge position of the reference samples of Fe foil, Fe_3_O_4_ and Fe_2_O_3_ as Fe^0+^, Fe^2.67+^, and Fe^3+^ references, respectively, for quantifying the Fe valence state in Fe-MOF_x_; it was confirmed that the calculated K-edge values were in a linear relationship with the valence state. The coordination shells were determined from the Fourier transformation of χ(k) data in k-space ranging from 0 to 12 Å^-1^, using Hamming windows (d_k_ = 1.0 Å^-1^). To obtain Morlet wavelet transforms (MWT) of the k^2^ weighted EXAFS spectra, the Fortran-based HAMA code was used.^[3]^

**Electrochemical Measurements**. To make the slurry, 3 mg of the Fe-MOF_x_ electrocatalyst and 7 mg of carbon black were dispersed in a mixture of 35 µL of Nafion (5%) solution and 965 µL of isopropyl alcohol, and sonicated for 30 min. 100 μL of this mixture was evenly loaded onto both sides of carbon cloth (0.5 × 0.5 cm^2^) and dried at room temperature. All measurements were performed on an electrochemical workstation VersaSTAT 3 using a standard three-electrode system. A graphite electrode and a saturated calomel electrode were used as the counter electrode and reference electrode, respectively. 1.0 M KOH solution was used as the electrolyte. LSV measurements were conducted at 5 mV s^-1^, and the reported potentials were provided with a reference to the reversible hydrogen electrode (RHE) according to the equation *E_vs RHE_ = E_vs SCE_ + 1.067* V in 1.0 M KOH. Polarization curves were corrected for the instrument response (IR) depending on the ohmic resistance of solution, by applying the equation E_Corrected_ = E_Raw_ – IR_s_*.* EIS measurements were carried out in the frequency range of 100 Hz to 0.01 Hz with a 5 mV amplitude. To evaluate the effective electrochemical active surface areas of the samples, the electrochemical capacitance (C_dl_) was obtained by CV measurements from 1.067 to 1.167 V (*vs. RHE*) at scanning rates of 2, 5, 10, 20, 50, 70 and 100 mV s^-1^. The overall water splitting was performed using a two-electrode system, where Fe-MOF_x_ electrodes served as both the cathode and anode. Electrolysis energy efficiency was calculated as the ratio between 1.48 V and the water splitting cell voltage:  $\varepsilon\cong(1.48 V)/E\_cell \times100\%$.

**Electrochemical measurements on an AEMWE device.** Fe-MOF_0.3_ was used as both the cathodic and anodic catalyst. The slurry of Fe-MOF_0.3_ catalysts was deposited onto Ni foam gas diffusion layers, with an estimated mass loading of 0.9 mg cm^-2^. Subsequently, the catalyst-coated layers were sandwiched together with an anion exchange membrane (Fuma, FAA-PK-130) to assemble an integrated AEMWE device. The anion exchange membrane was immersed into 1.0 M KOH solution for 24 h prior to being used to exchange Cl^-^ into OH^-^. The performance of AEMWE was evaluated by measuring the polarization curves from 1.4 V to 2.2 V. The stability of the AEMWEs was evaluated by applying the potential periodically (cycling between 1.6 V to 1.9 V, 5 h per 0.1 V).

***In situ* FTIR measurements.** The FTIR testing device consisted of two parts, including an IR spectrometer with a KBr beam splitter and various detectors (herein, a liquid-nitrogen-cooled MCT detector was used) and an IR microscope (Bruker INVENIO) with a 16× objective, which enabled measurements over a broad range of 15–4000 cm^−1^ and a high spectral resolution of 0.25 cm^−1^. Each high-resolution (2 cm^−1^) IR absorption spectrum was obtained by averaging 4 scans. The FTIR cell (EC-ATR) was supplied by Zhongyanhuanke Tec. The IR transmission window of the cell was made of a Si crystal. A constant potential was applied to the electrocatalyst electrode for 5 min, and then all IR spectral acquisitions were carried out. The background spectrum of the electrocatalyst electrode was acquired at an open-circuit voltage before each systemic OER measurement, and the measured potentials ranges of the OER were 0.8–1.5 V (versus RHE). A Chi600E workstation was used to control the measurements.

***In situ* Raman spectroscopy**. Raman spectra were measured on a HORIBA Scientific LabRAM HR Evolution spectrometer. A wavelength of He-Ne excitation laser is 532 nm. Spectra were recorded in the range of 300-1800 cm^-1^ with an acquisition time of 120 s and accumulation of 2 spectra. The electrochemical cell was supplied by Shanghai Chuxi in a horizontal configuration, allowing for irradiation from the top. A Chi600E workstation was used to control the measurements.

**DFT calculations.** First principles DFT calculations were carried out based on VASP.^[4-5]^ The Perdew–Burke–Ernzerhof (PBE) functional within generalized gradient approximation (GGA) was used to process the exchange–correlation, while the projector augmented-wave pseudopotential (PAW) was applied with a kinetic energy cut-off of 500 eV, which was utilized to describe the expansion of the electronic eigenfunctions. The Brillouin-zone integration was sampled by a Γ-centered 5 × 5 × 5 Monkhorst–Pack k-point. All atomic positions were fully relaxed until energy and force reached a tolerance of 1 × 10-5 eV and 0.03 eV/Å, respectively. The dispersion corrected DFT-D method was employed to consider the long-range interactions.

The Gibbs free energy change (ΔG) was calculated by computational hydrogen electrode (CHE) model as follows: ΔG = ΔE + ΔZPE – TΔS, where ΔE is the reaction energy obtained by the total energy difference between the reactant and product molecules absorbed on the catalyst surface and ΔS is the change in entropy for each reaction, ΔZPE is the zero-point energy correction to the Gibbs free energy. T stays for room temperature (298.15 K).


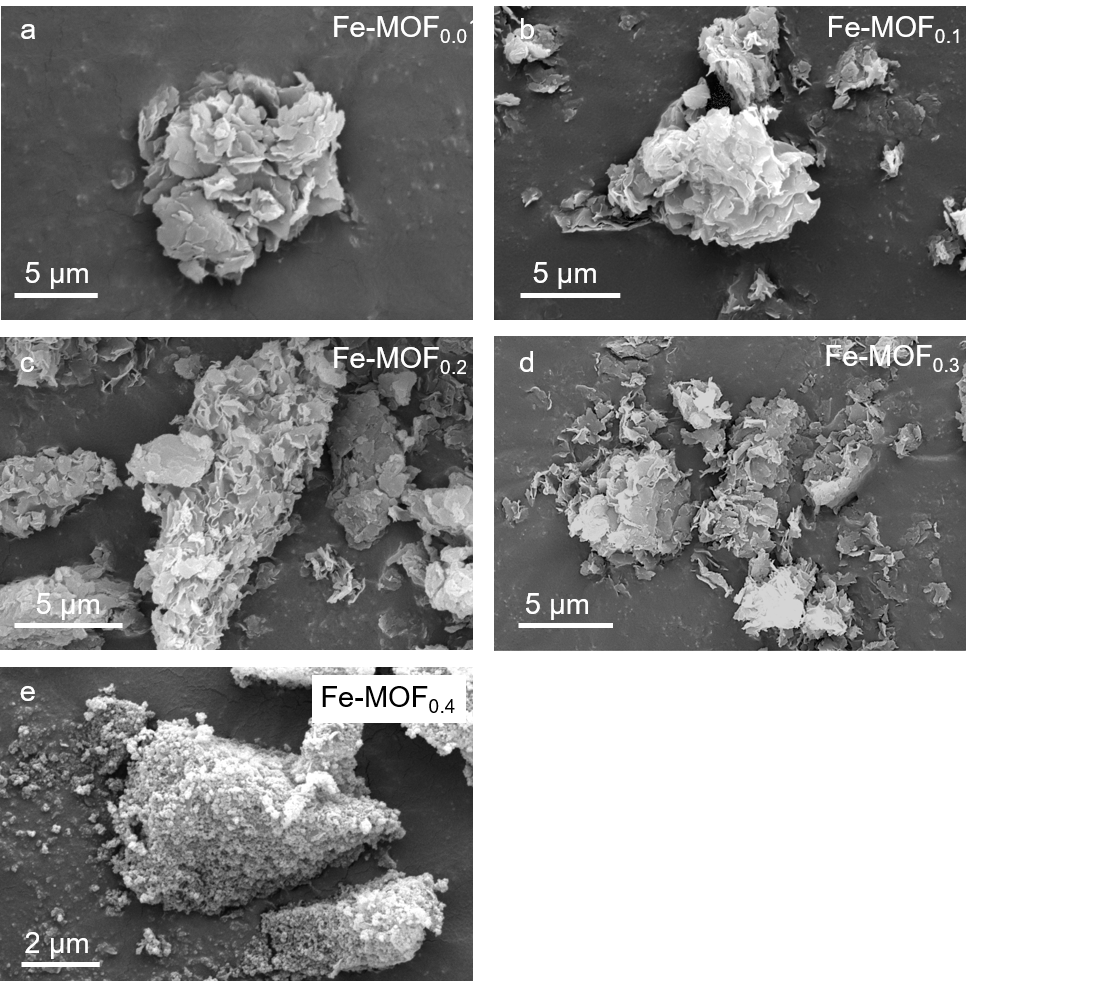


**Figure S1.** SEM images of a) Fe-MOF_0.0_, b) Fe-MOF_0.1_, c) Fe-MOF_0.2_, d) Fe-MOF_0.3_ and e) Fe-MOF_0.4_ catalysts.


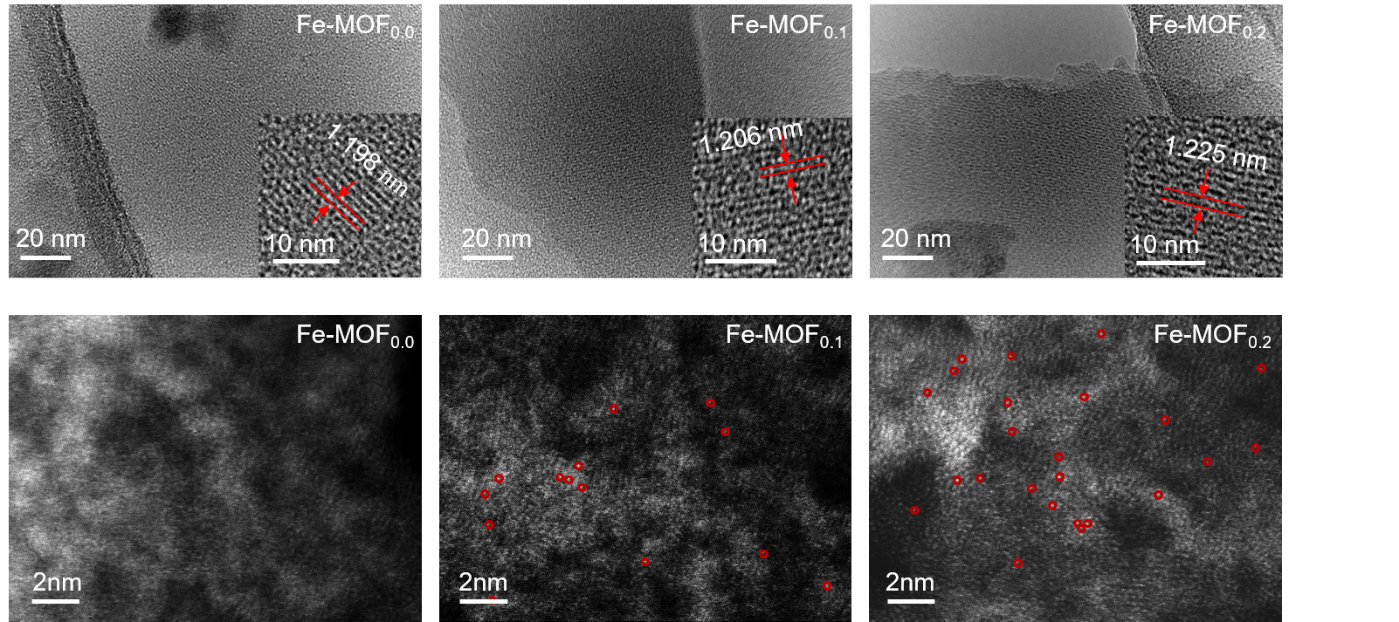


**Figure S2.** TEM images, and the corresponding lattice distances (shown in insets) of Fe-MOF_0.0_, Fe-MOF_0.1_, and Fe-MOF_0.2_ catalysts.


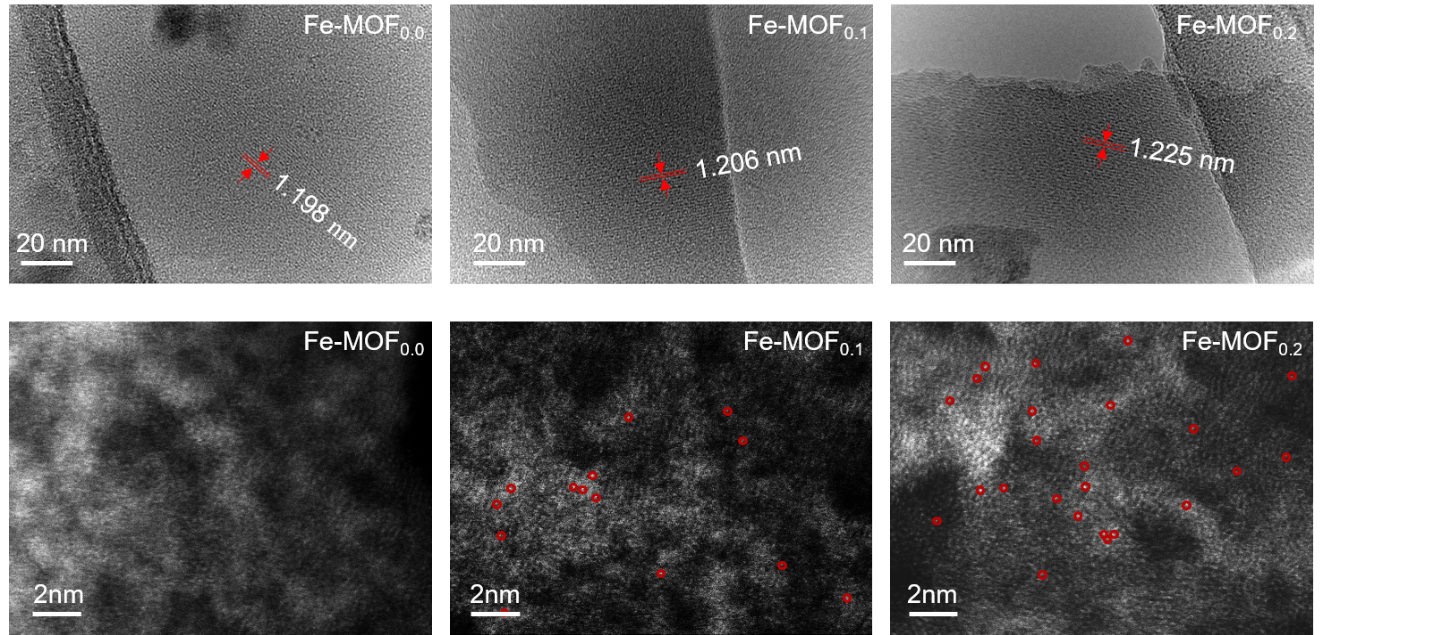


**Figure S3.** HAADF-STEM images of Fe-MOF_0.0_, Fe-MOF_0.1_, and Fe-MOF_0.2_ catalysts. Red circles illustrate the locations of bright white spots belonging to Fe single atoms.


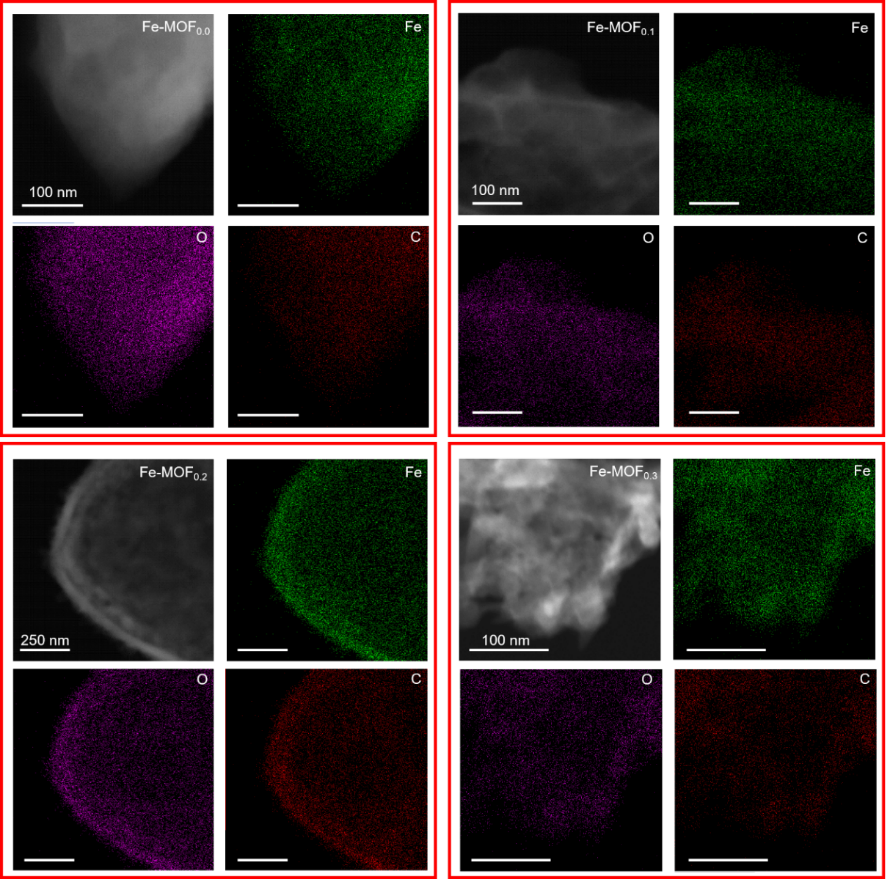


**Figure S4.** Elemental mapping of Fe, O and C performed on Fe-MOF_0.0_, Fe-MOF_0.1_, Fe-MOF_0.2_ and Fe-MOF_0.3_ catalysts; respective TEM images are also provided.


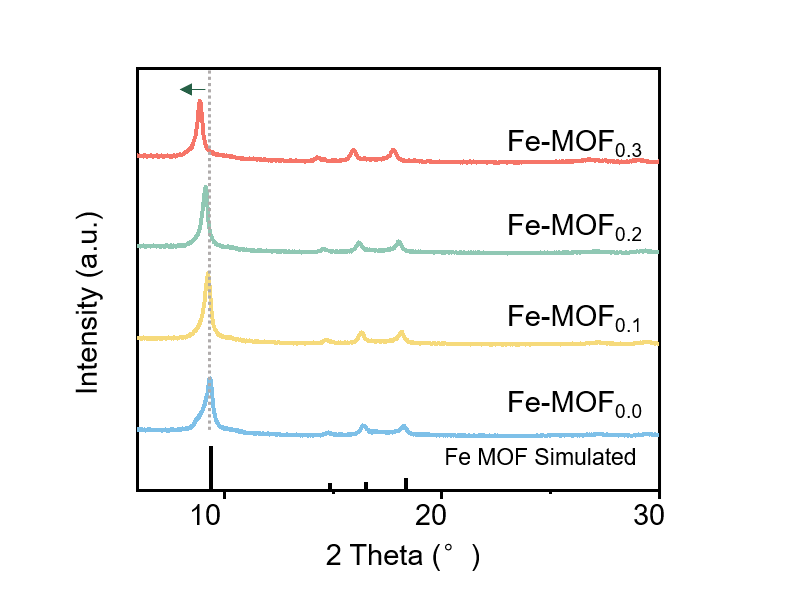


**Figure S5****.** XRD patterns of the four studied Fe-MOF_x_ catalysts, and the reference Fe-MOF_0.0_ at the bottom (line pattern) simulated from the single crystal data.


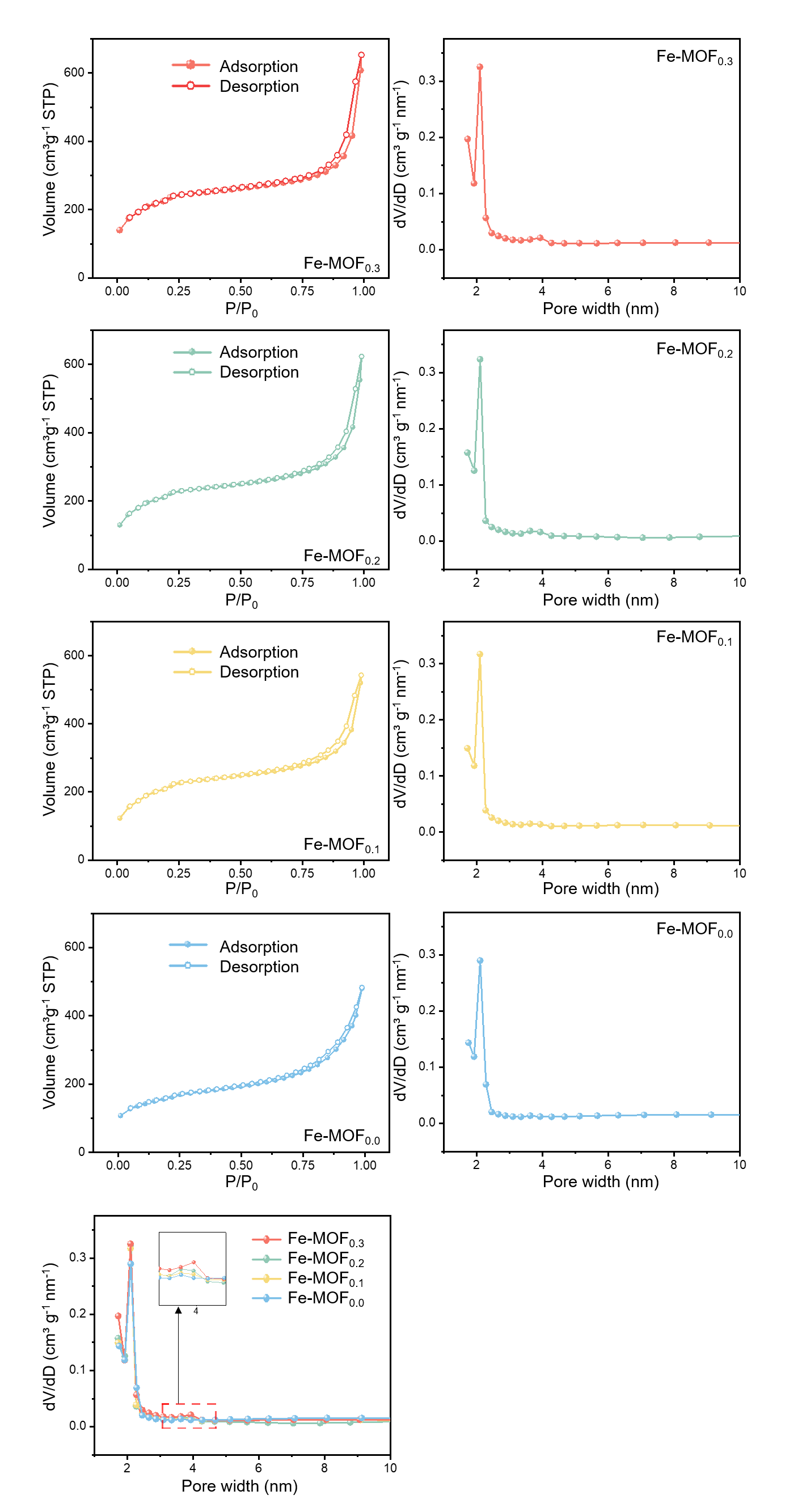


**Figure S6**. N_2_ sorption isotherms and pore size distributions of the four studied Fe-MOF_x_ catalysts.


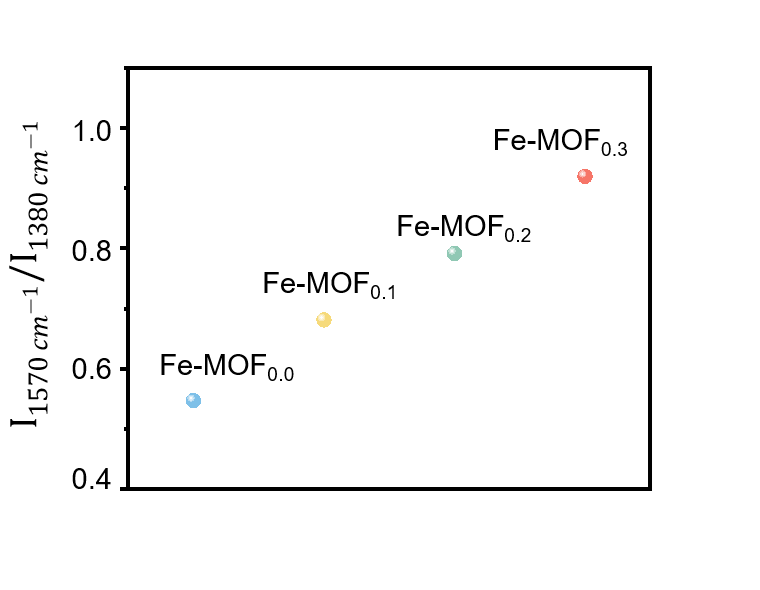


**Figure S7.** The intensity ratio of the FTIR bands at 1570 cm^-1^ and 1380 cm*^-^*^1^ (from Figure 1d).

**
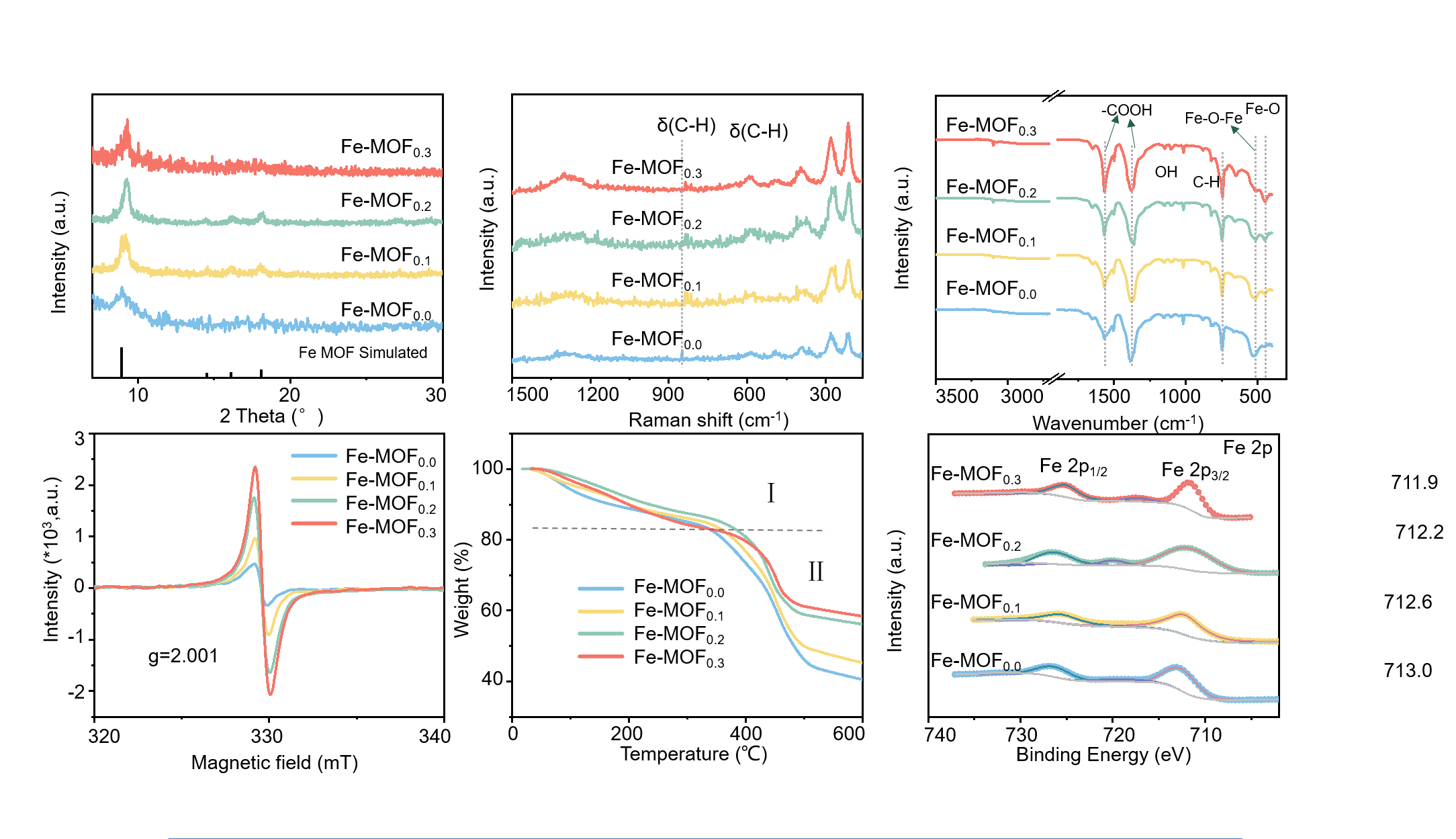
**

**Figure S8.** Thermogravimetric analysis of the four Fe-MOF_x_ catalysts.

**
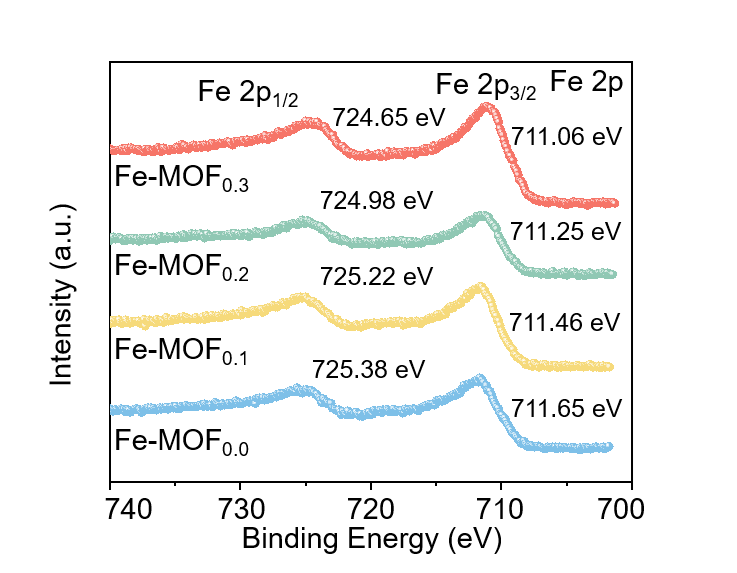
**

**Figure S9.** High-resolution XPS spectra of Fe 2p in the four Fe-MOF_x_ catalysts.


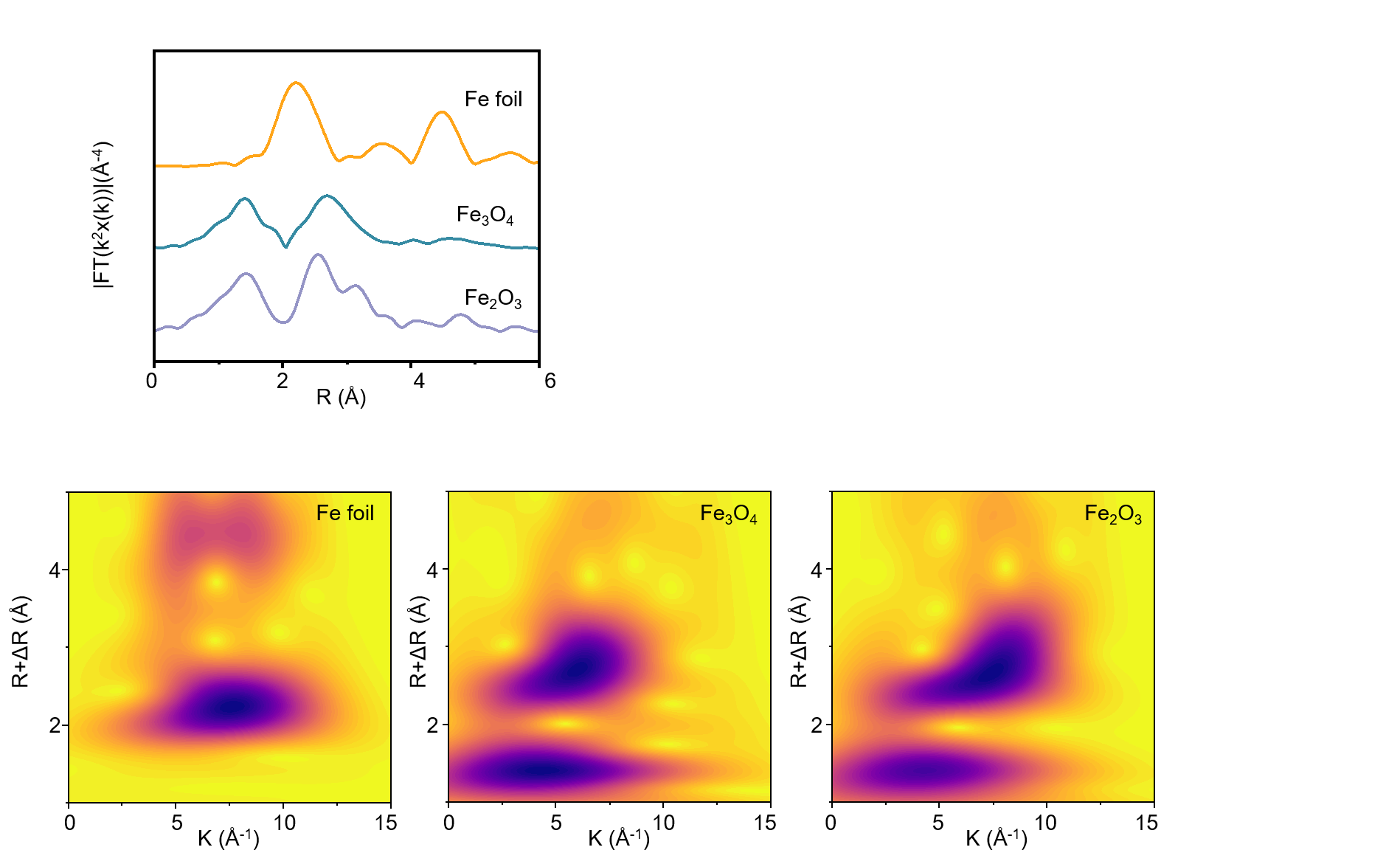


**Figure S10**. k^2^-weighted Fourier transform (FT) of the Fe K-edge EXAFS of the reference samples of Fe foil, Fe_3_O_4_ and Fe_2_O_3_.

**
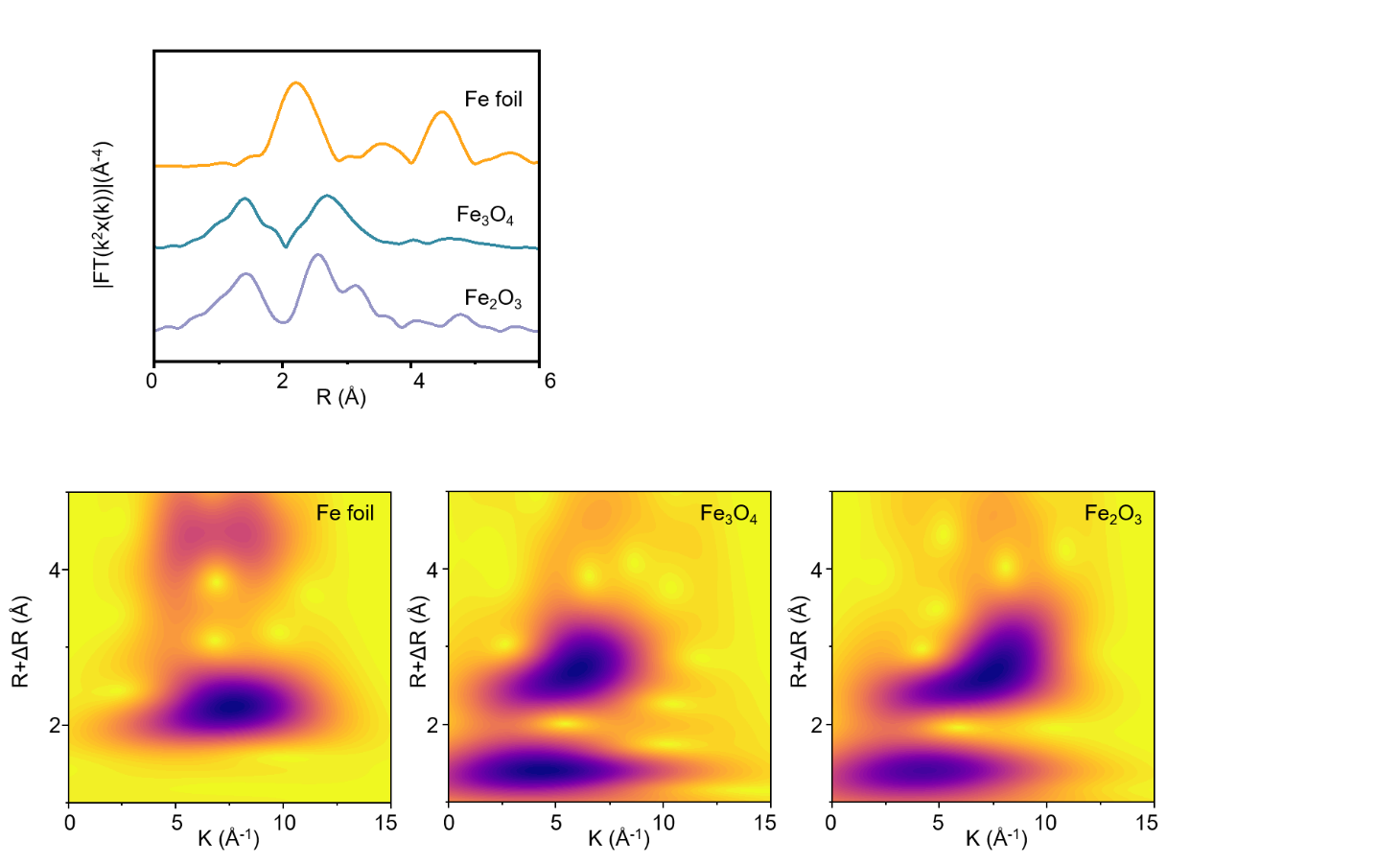
**

**Figure S11**. MWT images of Fe K-edge EXAFS for the reference samples of Fe foil, Fe_3_O_4_ and Fe_2_O_3_.


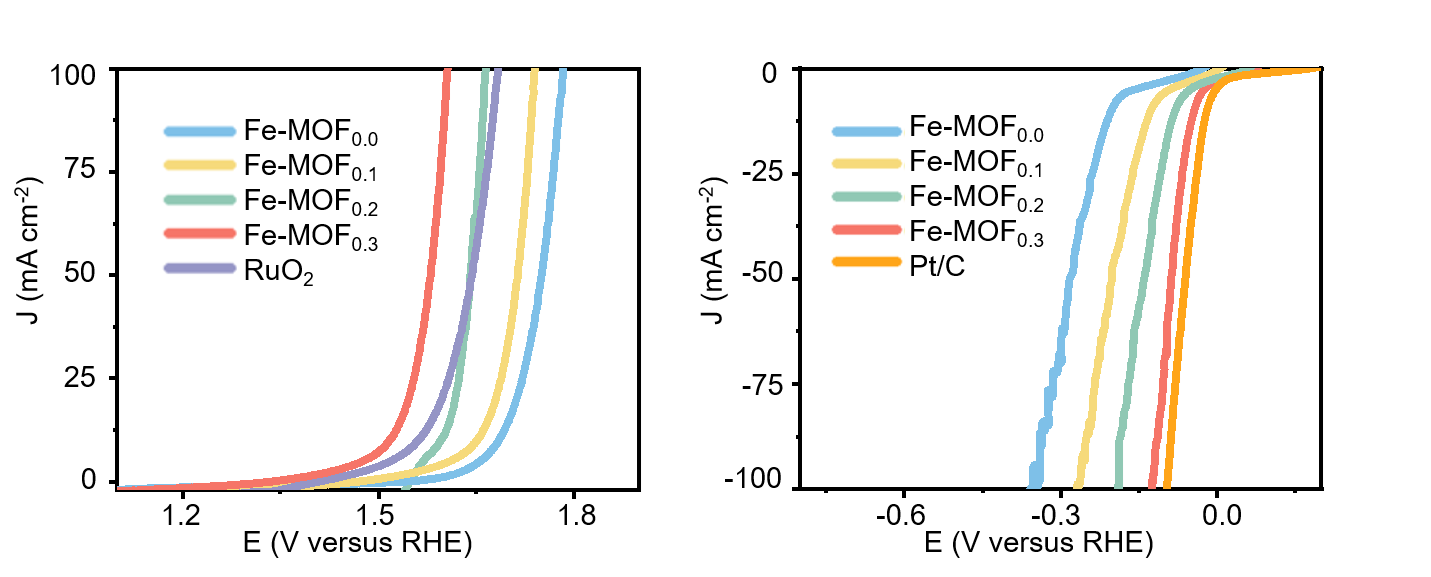


**Figure S12**. OER LSV curves of the four Fe-MOF_x_ electrodes, compared to the reference RuO_2_ electrode and recorded at 5 mV s^−1^.

**
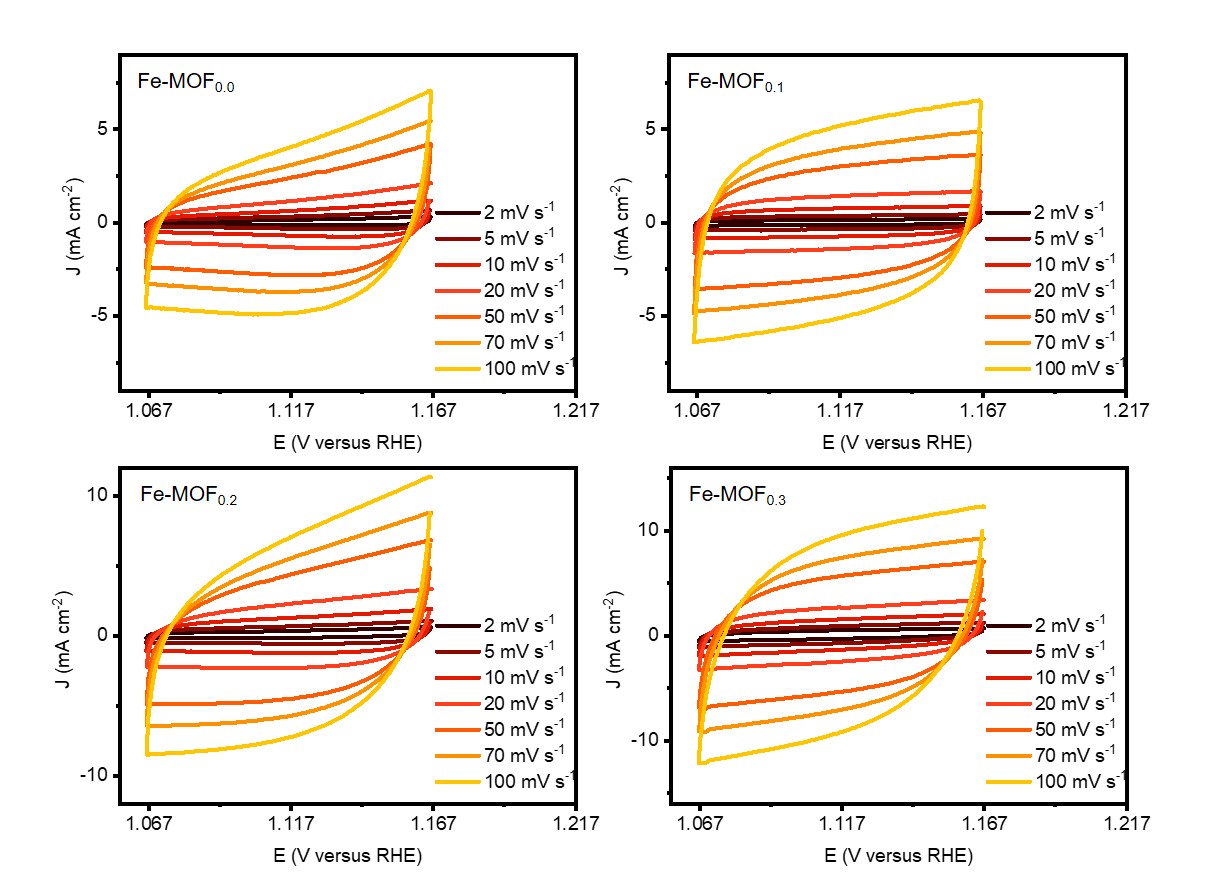
**

**Figure S13.** CV curves of the four Fe-MOF_x_ electrodes recorded in 1.0 M KOH at different scan rates.

**
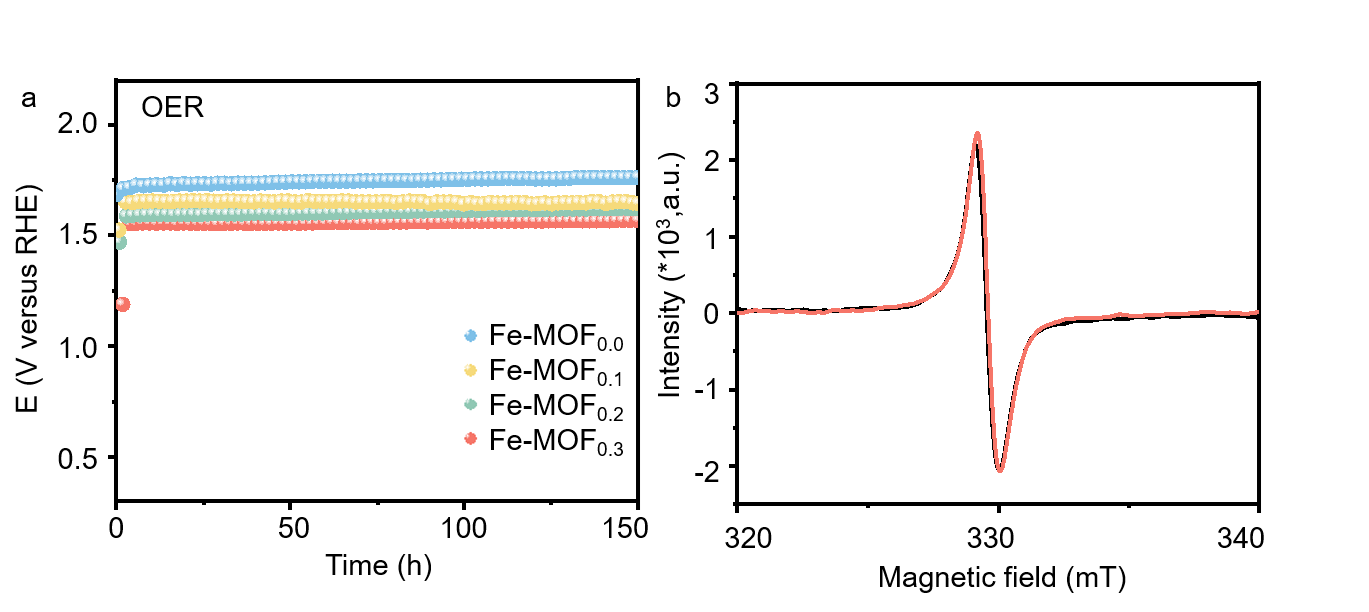
**

**Figure S14.** a) OER chronopotentiometry response of the four Fe-MOF_x_ electrodes at a current density of 10 mA cm^−2^. b) EPR spectra of pristine Fe-MOF_0.3_ (red curve) and of the same electrocatalyst after cycling for 150 h (black curve).


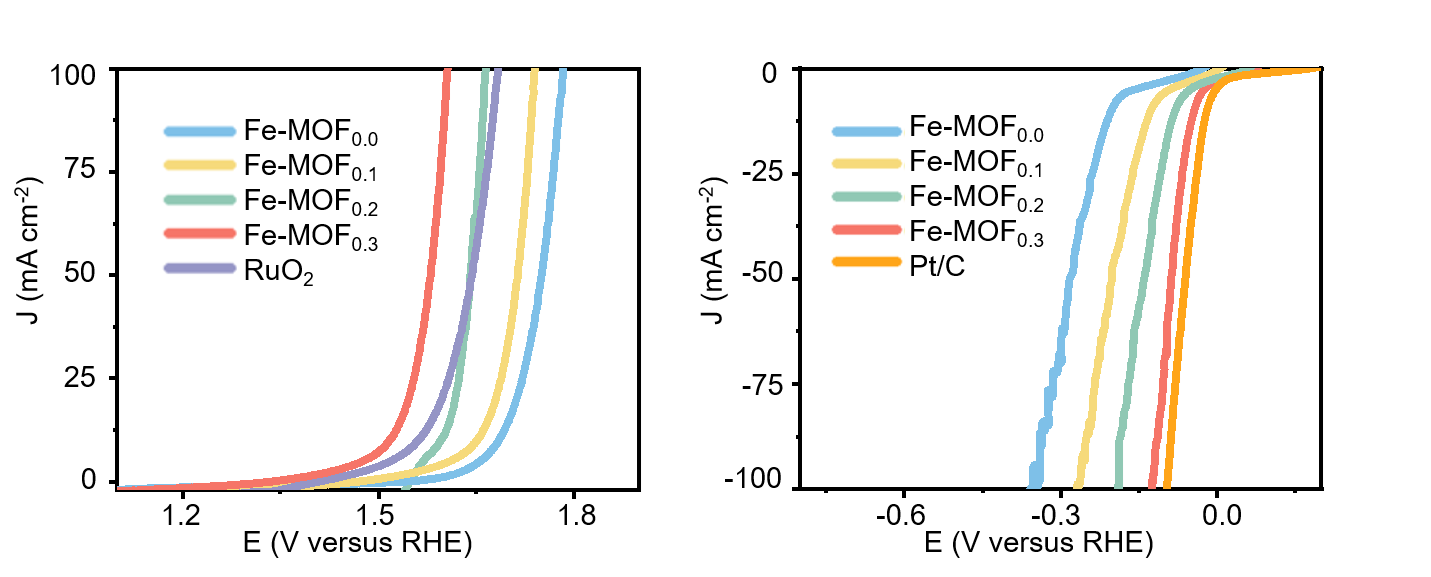


**Figure S15**. HER LSV curves of the four Fe-MOF_x_ electrodes, compared to the reference Pt/C electrode and recorded at 5 mV s^−1^.

**
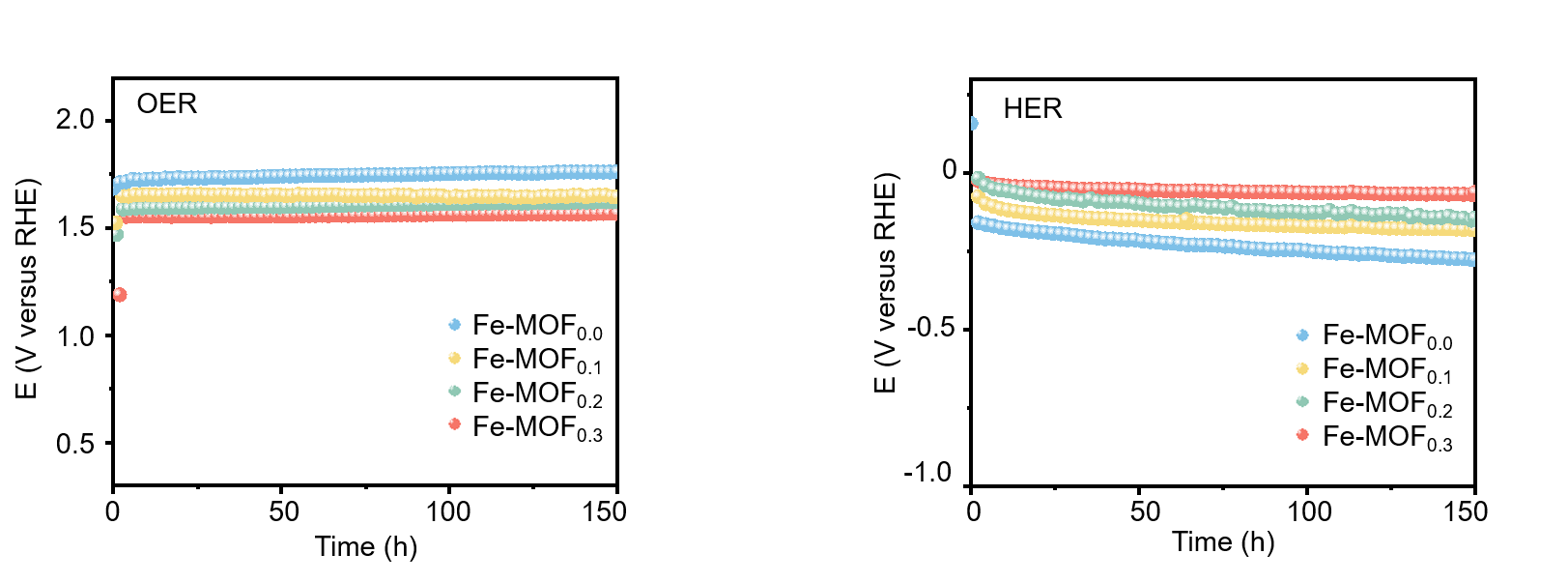
**

**Figure S16.** HER chronopotentiometry response of the four Fe-MOF_x_ electrodes at a current density of 10 mA cm^−2^.

**
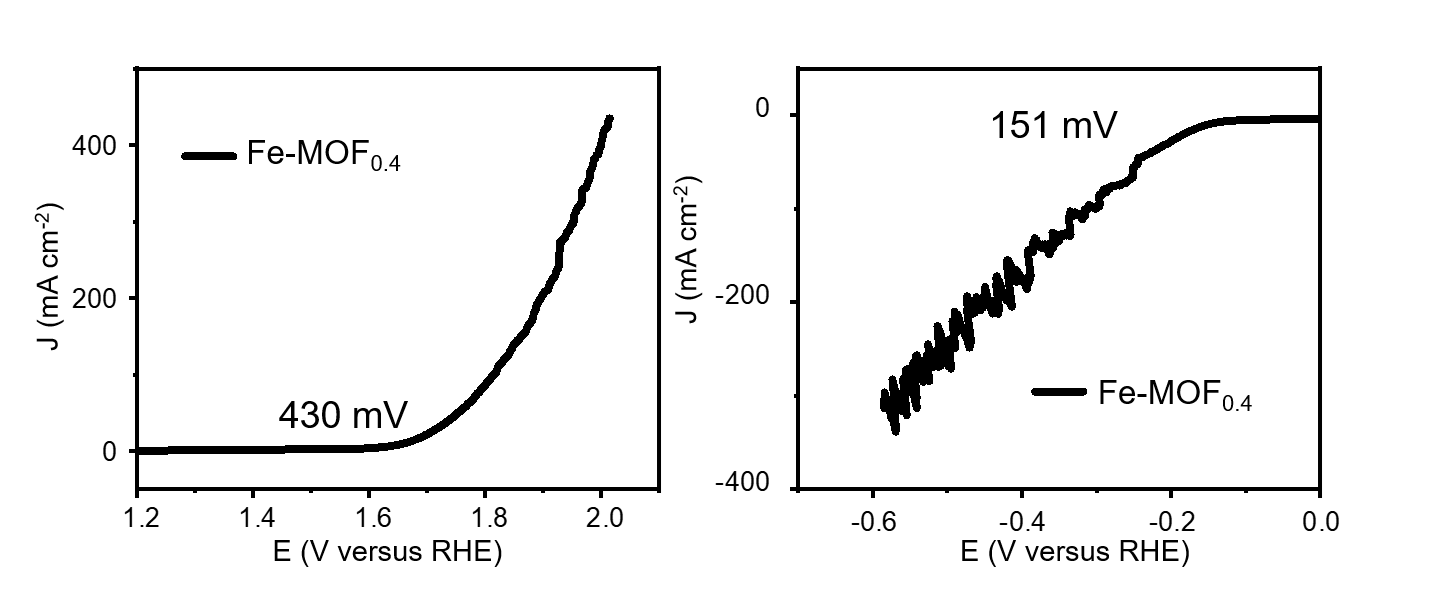
**

**Figure S17.** OER and HER LSV curves of the Fe-MOF_0.4_ electrodes at 5 mV s^−1^.


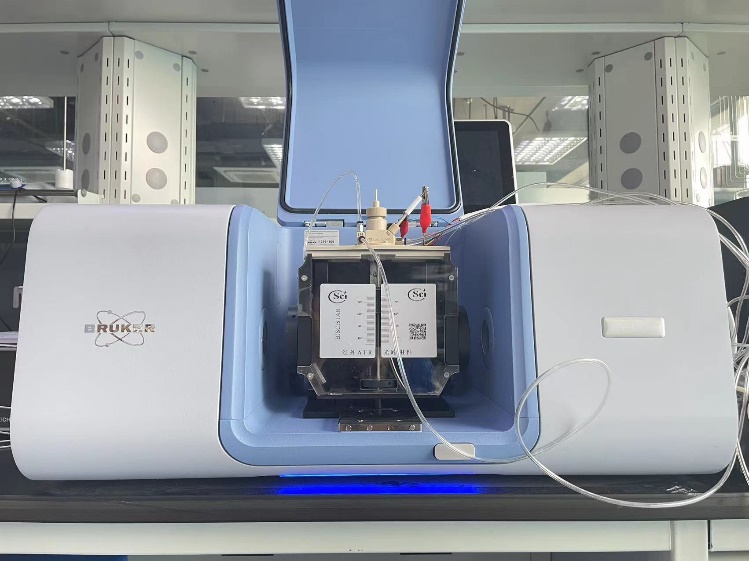

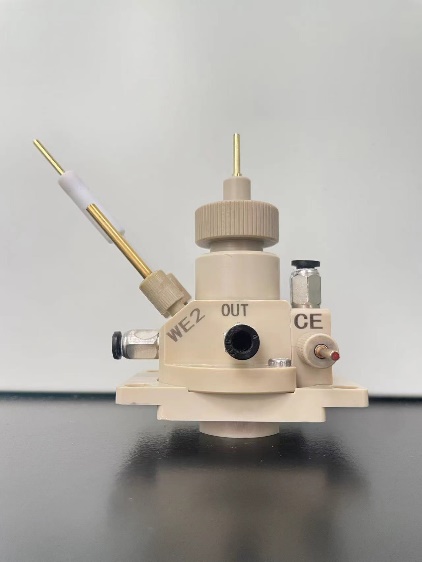


**Figure S18.** Photograph of the *in situ* FTIR testing system and the FTIR cell on the right.


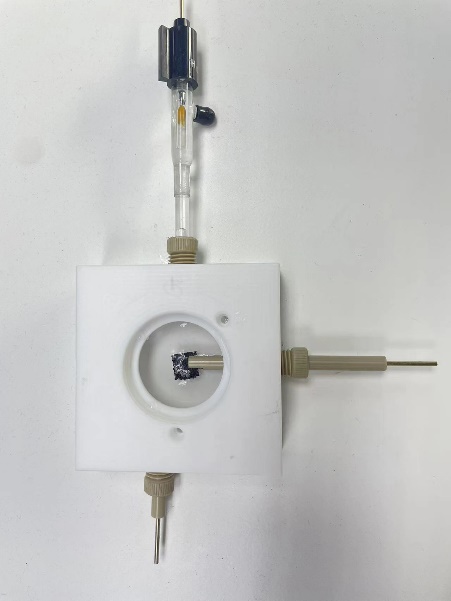

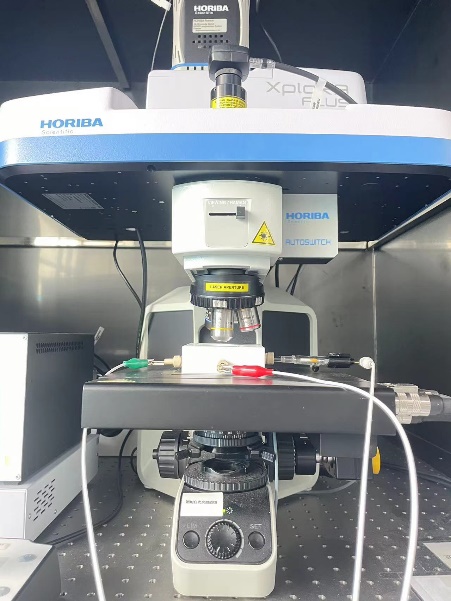


**Figure S19.** Photograph of the *in situ* Raman testing system and the cell on the left.


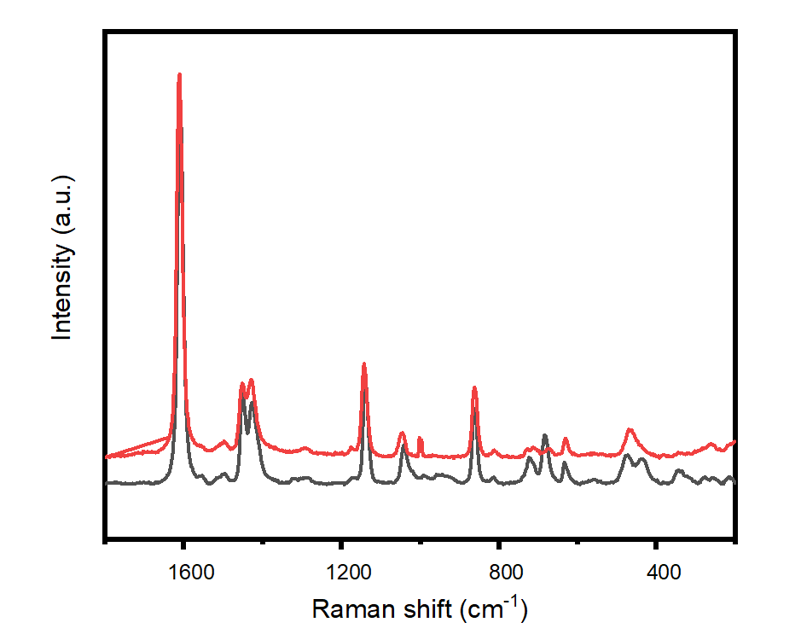


**Figure S20.** Raman spectra of pristine Fe-MOF_0.3_ (black curve) and of the same electrocatalyst after cycling for 6 h in the Raman cell in 1.0 M KOH (red curve).

**
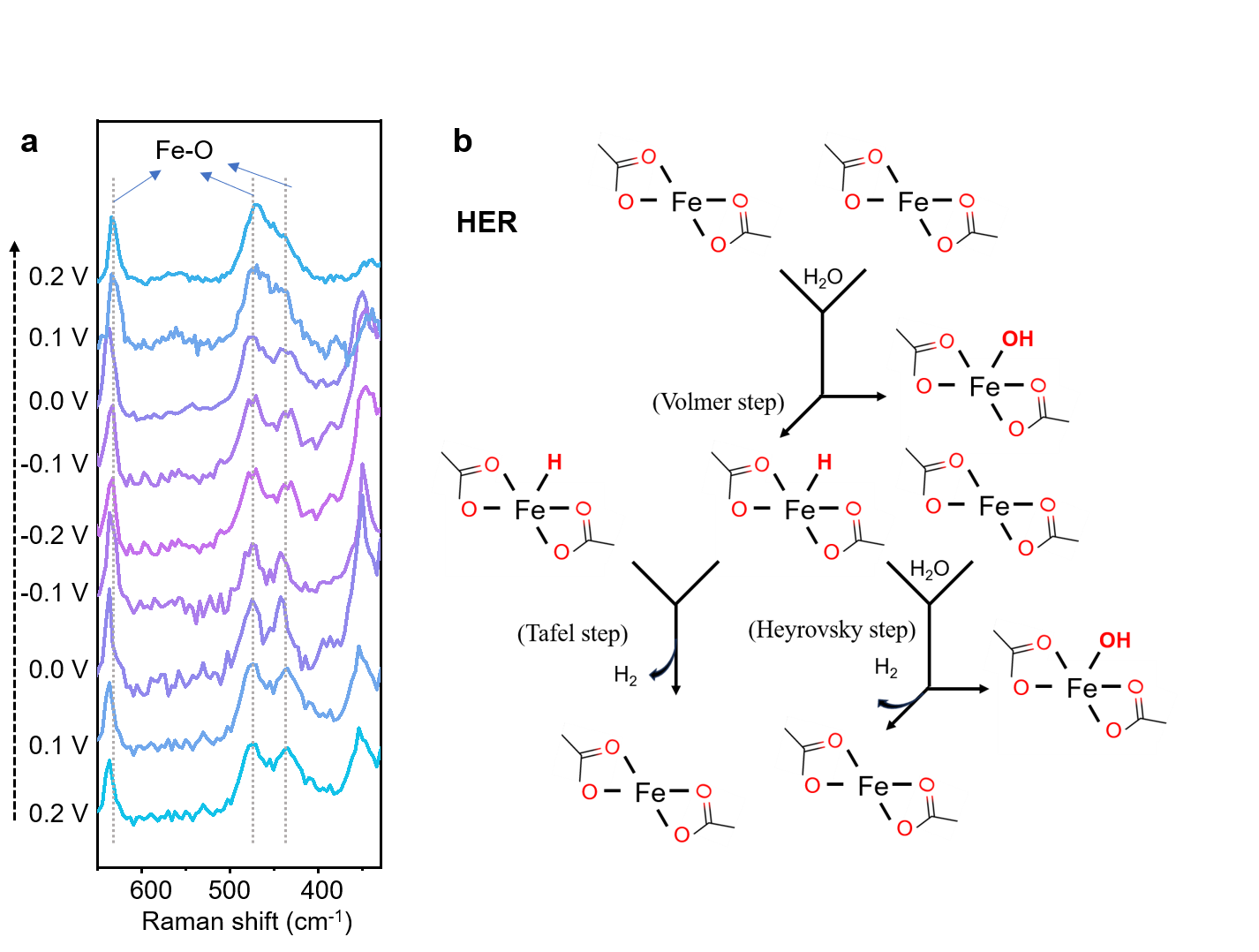
**

**Figure S21.** a) *In situ* Raman spectra recorded in the range of 650 – 330 cm^−1^ under various potentials (versus RHE) for Fe-MOF_0.3_ electrocatalyst during the HER process. All spectra were recorded during steady-state conditions; the laser excitation wavelength was 532 nm, and the solvent was 1.0 M KOH. b) Proposed electron transfer HER pathway for Fe-MOF_0.3_.

**
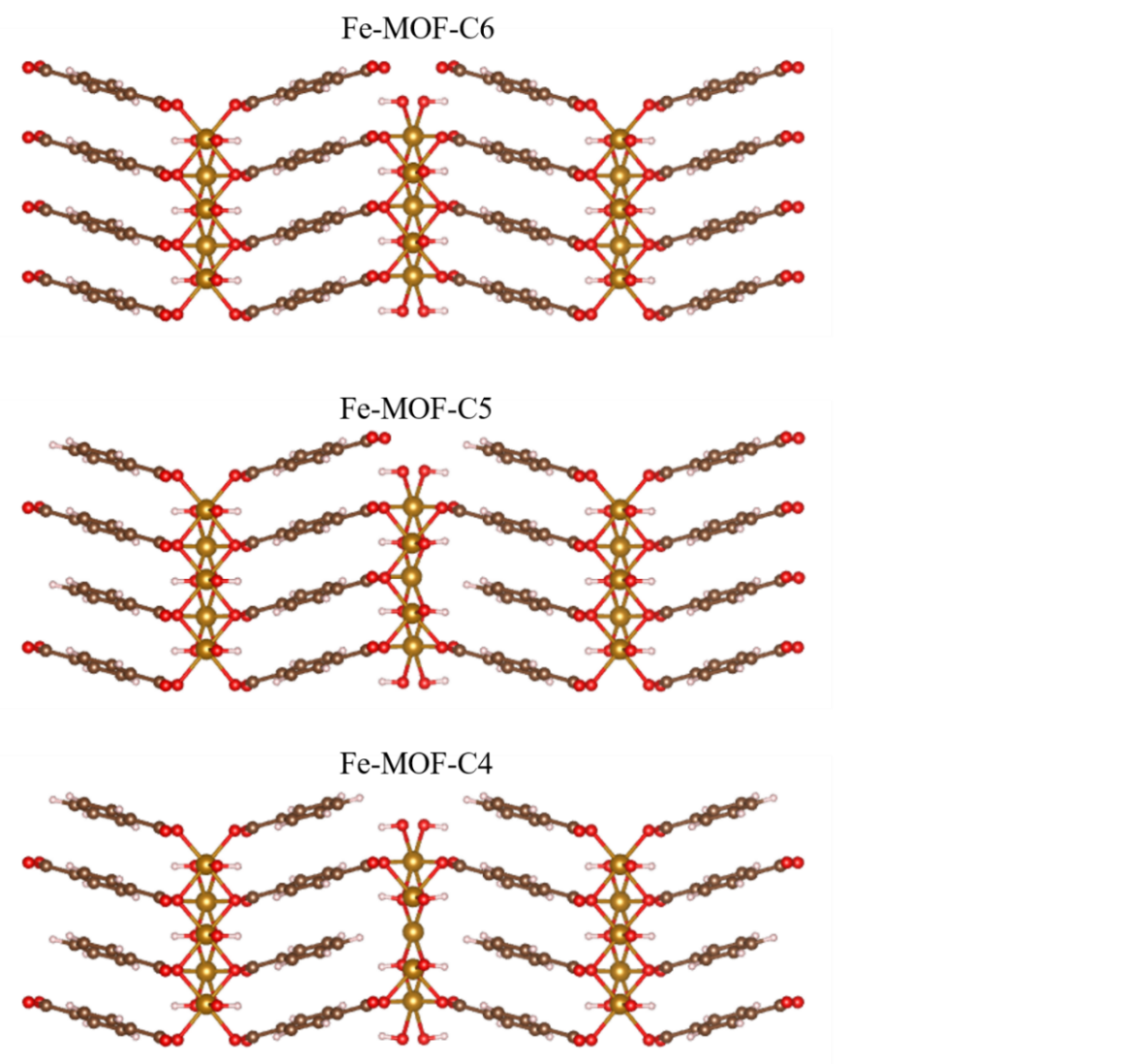
**

**Figure S22.** Optimized configurations of Fe-MOF-C6, Fe-MOF-C5 and Fe-MOF-C4 model structures used in DFT calculations.

**
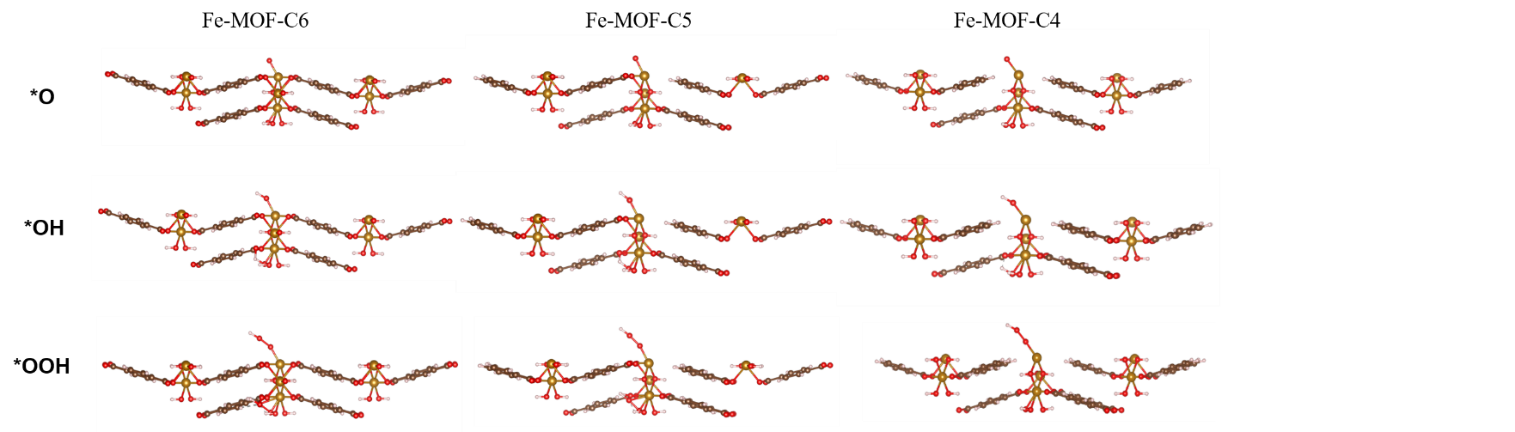
**

**Figure S23**. Optimized configurations of Fe-MOF-C6, and Fe-MOF-C5 and Fe-MOF-C4 model structures illustrating chemisorption of three intermediates (*O, *OH and *OOH) in the OER process in an alkaline solution.


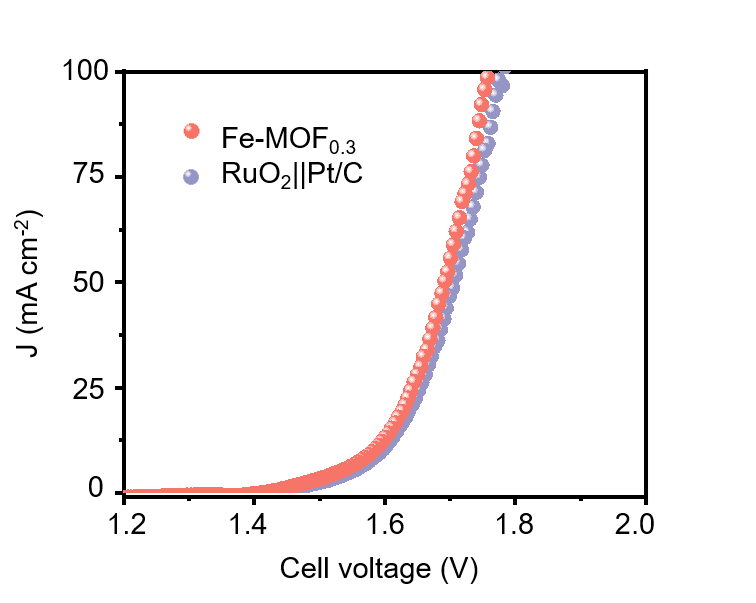


**Figure S24**. LSV responses of the water electrolyzers constructed from Fe-MOF_0.3_ (+,-) and RuO_2_(+)||Pt/C(-), recorded in 1.0 M KOH at a scan rate of 5 mV s^–1^.

**Table S1.** Room temperature ^57^Fe Mössbauer parameters for the Fe-MOF_x_ catalysts (related to **Figure 1f**).

| Component |  | IS  (mm/s) | QS  (mm/s) | Г/2  (mm/s) | Area | Assignments |
| --- | --- | --- | --- | --- | --- | --- |
| Fe-MOF_0.0_ | Doublet 1 | 0.39 | 0.92 | 0.25 | 0.957 | High-Spin Fe^Ⅲ^ |
|  | Doublet 2 | 0.98 | 2.65 | 0.14 | 0.043 | High-Spin Fe^Ⅱ^ |
| Fe-MOF_0.1_ | Doublet 1 | 0.4 | 0.89 | 0.25 | .866 | High-Spin Fe^Ⅲ^ |
|  | Doublet 2 | 1.03 | 2.58 | 0.23 | 0.134 | High-Spin Fe^Ⅱ^ |
| Fe-MOF_0.2_ | Doublet 1 | 0.4 | 0.89 | 0.26 | 0.783 | High-Spin Fe^Ⅲ^ |
|  | Doublet 2 | 1.06 | 2.46 | 0.24 | 0.217 | High-Spin Fe^Ⅱ^ |
| Fe-MOF_0.3_ | Doublet 1 | 0.38 | 0.93 | 0.28 | 0.714 | High-Spin Fe^Ⅲ^ |
|  | Doublet 2 | 1.15 | 2.29 | 0.19 | 0.286 | High-Spin Fe^Ⅱ^ |

Г/2: full width at half maximum​

**Table S2.** Fe K-edge EXAFS analysis for the Fe-MOF_x_ catalysts.

|  | Path | CN | R (Å) | σ^2^ (Å^2^) | ∆E_0_ (eV) | R-factor |
| --- | --- | --- | --- | --- | --- | --- |
| Fe-MOF_0.0_  Fe-MOF_0.1_ | Fe-O | 5.95 | 1.98 | 0.0063 | 5.637  0.974 | 0.0095  0.0156 |
|  | Fe-O | 5.35 | 1.99 | 0.0046 |  |  |
| Fe-MOF_0.2_  Fe-MOF_0.3_ | Fe-O | 4.88 | 1.97 | 0.0080 | 7.712  4.107 | 0.0097  0.0093 |
|  | Fe-O | 4.46 | 1.98 | 0.0082 |  |  |

N, coordination number; R, distance between absorber and backscatter atoms; σ^2^, Debye–Waller factor to account for both thermal and structural disorders; ΔE_0_(eV), inner potential correction to account for the difference in the inner potential between the sample and the reference compound; R-factor (%) indicates the goodness of the fit. The amplitude reduction factor (S_0_^2^) was set to 0.9 during the fitting process.

**Table S3.** ICP-OES data measured on the pristine Fe-MOF_x_ and on the same electrocatalyst after cycling for 150 h in 1.0 M KOH.

| Fe content (wt%) | Fe-MOF_0.0_ | Fe-MOF_0.1_ | Fe-MOF_0.2_ | Fe-MOF_0.3_ |
| --- | --- | --- | --- | --- |
| Pristine | 9.82 | 10.16 | 11.34 | 12.28 |
| After cycling for 150 h | 8.81 | 9.35 | 10.84 | 11.98 |

**Table S4.** A comparison of the performance of the optimized Fe-MOF_0.3_ catalyst produced in this work with the previously reported MOF-based/derived electrocatalysts for OER.

| Electrocatalyst | Electrolyte | Over-potential at 10 mAcm^-2^ (mV) | Ref. |
| --- | --- | --- | --- |
| Ni_2_P@C/G | 1.0 M KOH | 285 | ^[6]^ |
| Co_3_Fe_7_@Fe_2_N/rGO | 1.0 M KOH | 371 | ^[7]^ |
| CoC_x_/FeCo@C/rGO | 1.0 M KOH | 390 | ^[8]^ |
| Co_2_P/Mo_2_C/Mo_3_Co_3_C@C | 1.0 M KOH | 362 | ^[9]^ |
| Co/Co_9_S_8_@SNGS | 1.0 M KOH | 290 | ^[10]^ |
| NiFe@C | 1.0 M KOH | 281 | ^[11]^ |
| FeNi/NiFe_2_O_4_@NC | 1.0 M KOH | 316 | ^[12]^ |
| NiCo_2_S_4_@CC | 1.0 M KOH | 370 | ^[13]^ |
| N–CoO@CoP | 1.0 M KOH | 332 | ^[14]^ |
| Cu_3_P@C-120 | 1.0 M KOH | 300 | ^[15]^ |
| MnCo_2_O_4_@Co_3_O_4_ | 1.0 M KOH | 280 | ^[16]^ |
| **Fe-MOF_0.3_** | **1.0 M KOH** | **259** | **This work** |

**Table S5.** A comparison of the optimized Fe-MOF_0.3_ catalyst produced in this work with the previously reported MOF-based/derived electrocatalysts for HER.

| Electrocatalyst | Electrolyte | Overpotential at 10 mA cm^-2^ (mV) | Ref. |
| --- | --- | --- | --- |
| NCT@CoP@MoS_2_ | 1.0 M KOH | 195 | ^[17]^ |
| Zn_0.3_Co_2.7_S_4_ | 0.5 M H_2_SO_4_ | 80 | ^[18]^ |
| CoP/NCNHP | 1.0 M KOH | 115 | ^[19]^ |
| Co–Ni@Fe–Cu-GR NCs | 0.5 M H_2_SO_4_ | 288 | ^[20]^ |
| CoP@FeCoP/NC | 1.0 M KOH | 141 | ^[21]^ |
| Fe/Zn-CoP | 1.0 M KOH | 75 | ^[22]^ |
| FeCo-FeCoP@C@NCCs | 1.0 M KOH | 93 | ^[23]^ |
| Fe_3_S_4_ /MIL-53 | 1.0 M KOH | 92 | ^[24]^ |
| Fe(OH)_x_@Cu-MOF | 1.0 M KOH | 112 | ^[25]^ |
| FeBHT | pH 1.3 H_2_SO_4_ | 173 | ^[26]^ |
| CoTHT | 1.0 M KOH | 143 | ^[27]^ |
| **Fe-MOF_0.3_** | **1.0 M KOH** | **36** | **This work** |

**Table S6.** The intensities of Raman bands at 437 cm^-1^, 469 cm*^-^*^1^ and 634 cm*^-^*^1^ assigned to the Fe-O bond with symmetric vibration (F_2g_), asymmetric bending(T_2g_) and Fe-O symmetric stretching (A_1g_).

| Intensity | 437 cm^-1^ | 469 cm^-1^ | 634cm^-1^ | $I_{{437 cm}^{-1}}/(I_{{437 cm}^{-1}+}I_{{469 cm}^{-1}+}I_{{634 cm}^{-1}})$ |
| --- | --- | --- | --- | --- |
| Fe-MOF_0.3_ | 331.55 | 241.54 | 256.9 | 29.1% |

**Table S7.** A comparison of the two-electrode electrolyzer based on Fe-MOF_0.3_ (+,−) with the previously reported MOF-based/derived electrocatalysts.

| Electrocatalyst | Electrolyte | Cell voltage at 10 mA cm^-2^(V) | Ref. |
| --- | --- | --- | --- |
| PBAs@PANI | 1.0 M KOH | 1.73 | ^[28]^ |
| Co/NBC-900 | 1.0 M KOH | 1.68 | ^[29]^ |
| NiO/Co_3_O_4_ NCMC | 1.0 M KOH | 1.63 | ^[30]^ |
| Co-NC@Mo_2_C | 1.0 M KOH | 1.69 | ^[31]^ |
| NiFe-P@GNS | 1.0 M KOH | 1.58 | ^[32]^ |
| Co-NCNTFs//NF | 1.0 M KOH | 1.62 | ^[33]^ |
| Co_0.75_Fe_0.25_-NC | 1.0 M KOH | 1.68 | ^[34]^ |
| H-CoS*_x_*@NiFe LDH | 1.0 M KOH | 1.59 | ^[35]^ |
| Co-Mo-P@NCNS | 1.0 M KOH | 1.58 | ^[36]^ |
| CoP*_x_*@CNS | 1.0 M KOH | 1.63 | ^[37]^ |
| RuO_2_/Co_3_O_4_-RuCo@NC | 0.5 M H_2_SO_4_ | 1.66 | ^[38]^ |
| **Fe-MOF_0.3_** | **1.0 M KOH** | **1.58** | **This work** |

**References**

[1] D. C. Koningsberger, R. Prins, **1987**.

[2] B. Ravel, M. Newville, *J. Synchrotron Radiat.* **2005**, *12* (4), 537.

[3] C. Mikutta, R. Kretzschmar, *Environ Sci. Tech.* **2011**, *45* (22), 9550.

[4] G. Henkelman, B. P. Uberuaga, H. Jónsson, *J. Chem. Phys.* **2000**, *113* (22), 9901.

[5] G. Kresse, J. Furthmüller, *Comput. Mater. Sci.* **1996**, *6* (1), 15.

[6] M. Wang, M. Lin, J. Li, L. Huang, Z. Zhuang, C. Lin, L. Zhou, L. Mai, *Chem. Commun.* **2017**, *53* (59), 8372.

[7] D. Liang, H. Zhang, X. Ma, S. Liu, J. Mao, H. Fang, J. Yu, Z. Guo, T. Huang, *Mater. Today Energy* **2020**, *17*, 100433.

[8] H. Fang, T. Huang, Y. Sun, B. Kang, D. Liang, S. Yao, J. Yu, M. M. Dinesh, S. Wu, J. Y. Lee, *J. Catal.* **2019**, *371*, 185.

[9] X. Li, X. Wang, J. Zhou, L. Han, C. Sun, Q. Wang, Z. Su, *J. Mater. Chem. A* **2018**, *6* (14), 5789.

[10] X. Zhang, S. Liu, Y. Zang, R. Liu, G. Liu, G. Wang, Y. Zhang, H. Zhang, H. Zhao, *Nano Energy* **2016**, *30*, 93.

[11] Y. Feng, X.-Y. Yu, U. Paik, *Sci. Rep.* **2016**, *6* (1), 34004.

[12] Y. Ma, X. Dai, M. Liu, J. Yong, H. Qiao, A. Jin, Z. Li, X. Huang, H. Wang, X. Zhang, *ACS Appl. Mater. Interfaces* **2016**, *8* (50), 34396.

[13] D. Wang, L. Tian, J. Huang, D. Li, J. Liu, Y. Xu, H. Ke, Q. Wei, *Electrochim. Acta* **2020**, *334*, 135636.

[14] M. Lu, L. Li, D. Chen, J. Li, N. Klyui, W. Han, *Electrochim. Acta* **2020**, *330*, 135210.

[15] J. Rong, J. Xu, F. Qiu, Y. Zhu, Y. Fang, J. Xu, T. Zhang, *Adv. Mater. Interfaces* **2019**, *6* (14), 1900502.

[16] J.-J. Zhou, X. Han, K. Tao, Q. Li, Y.-L. Li, C. Chen, L. Han, *Chem. Eng. J.* **2018**, *354*, 875.

[17] C.-L. Zhang, Y. Xie, J.-T. Liu, F.-H. Cao, H.-P. Cong, H. Li, *Chem. Eng. J.* **2021**, *419*, 129977.

[18] Z.-F. Huang, J. Song, K. Li, M. Tahir, Y.-T. Wang, L. Pan, L. Wang, X. Zhang, J.-J. Zou, *J. Am. Chem. Soc.* **2016**, *138* (4), 1359.

[19] Y. Pan, K. Sun, S. Liu, X. Cao, K. Wu, W.-C. Cheong, Z. Chen, Y. Wang, Y. Li, Y. Liu, D. Wang, Q. Peng, C. Chen, Y. Li, *J. Am. Chem. Soc.* **2018**, *140* (7), 2610.

[20] M. Fu, G. Ning, J. Liu, Q. Zhang, Y. Sun, X. Fan, H. Wang, H. Lu, Y. Zhang, H. Wang, *Inter. J. Hydrogen Energy* **2021**, *46* (29), 15124.

[21] J. Shi, F. Qiu, W. Yuan, M. Guo, Z.-H. Lu, *Chem. Eng. J.* **2021**, *403*, 126312.

[22] P. Wang, X. Liu, Y. Yan, J. Cao, J. Feng, J. Qi, *Catal. Sci. Tech.* **2020**, *10* (5), 1395.

[23] Y. Li, S. Li, J. Hu, Y. Zhang, Y. Du, X. Han, X. Liu, P. Xu, *J. Energy Chem.* **2021**, *53*, 1.

[24] D.-D. Huang, S. Li, Y.-P. Wu, J.-H. Wei, J.-W. Yi, H.-M. Ma, Q.-C. Zhang, Y.-L. Liu, D.-S. Li, *Chem. Commun.* **2019**, *55* (31), 4570.

[25] W. Cheng, H. Zhang, D. Luan, X. W. Lou, *Sci. Adv.* **2021**, *7* (18), eabg2580.

[26] C. A. Downes, A. J. Clough, K. Chen, J. W. Yoo, S. C. Marinescu, *ACS Appl. Mater. Interfaces* **2018**, *10* (2), 1719.

[27] K. Chen, C. A. Downes, E. Schneider, J. D. Goodpaster, S. C. Marinescu, *ACS Appl. Mater. Interfaces* **2021**, *13* (14), 16384.

[28] L. Zhang, T. Meng, B. Mao, D. Guo, J. Qin, M. Cao, *RSC Adv.* **2017**, *7* (80), 50812.

[29] M. R. Liu, Q. L. Hong, Q. H. Li, Y. Du, H. X. Zhang, S. Chen, T. Zhou, J. Zhang, *Adv. Funct. Mater.* **2018**, *28* (26), 1801136.

[30] X. Wei, Y. Zhang, H. He, D. Gao, J. Hu, H. Peng, L. Peng, S. Xiao, P. Xiao, *Chem. Commun.* **2019**, *55* (46), 6515.

[31] Q. Liang, H. Jin, Z. Wang, Y. Xiong, S. Yuan, X. Zeng, D. He, S. Mu, *Nano Energy* **2019**, *57*, 746.

[32] D. Yang, Z. Su, Y. Chen, Y. Lu, B. Yu, K. Srinivas, B. Wang, W. Zhang, *J. Mater. Chem. A* **2020**, *8* (42), 22222.

[33] Q. Yuan, Y. Yu, Y. Gong, X. Bi, *ACS Appl. Mater. Interfaces* **2019**, *12* (3), 3592.

[34] X. Feng, X. Bo, L. Guo, *J. Power Sources* **2018**, *389*, 249.

[35] Y. J. Lee, S.-K. Park, *Small* **2022**, *18* (16), 2200586.

[36] N. Li, Y. Guan, Y. Li, H. Mi, L. Deng, L. Sun, Q. Zhang, C. He, X. Ren, *J. Mater. Chem. A* **2021**, *9* (9), 5868.

[37] C.-C. Hou, L. Zou, Y. Wang, Q. Xu, *Angew. Chem. Int. Ed.* **2020**, *59* (48), 21360.

[38] Z. Fan, J. Jiang, L. Ai, Z. Shao, S. Liu, *ACS Appl. Mater. Interfaces* **2019**, *11* (51), 47894.
